# Supplementary material for: Fate specification is spatially intermingled across planarian stem cells
Source: Nat Commun. 2023 Nov 16;14:7422. doi: 10.1038/s41467-023-43267-2 (PMC10654723; doi:10.1038/s41467-023-43267-2)
Supplement: Supplementary file 1 — Supplementary Information [file 41467_2023_43267_MOESM1_ESM.pdf]

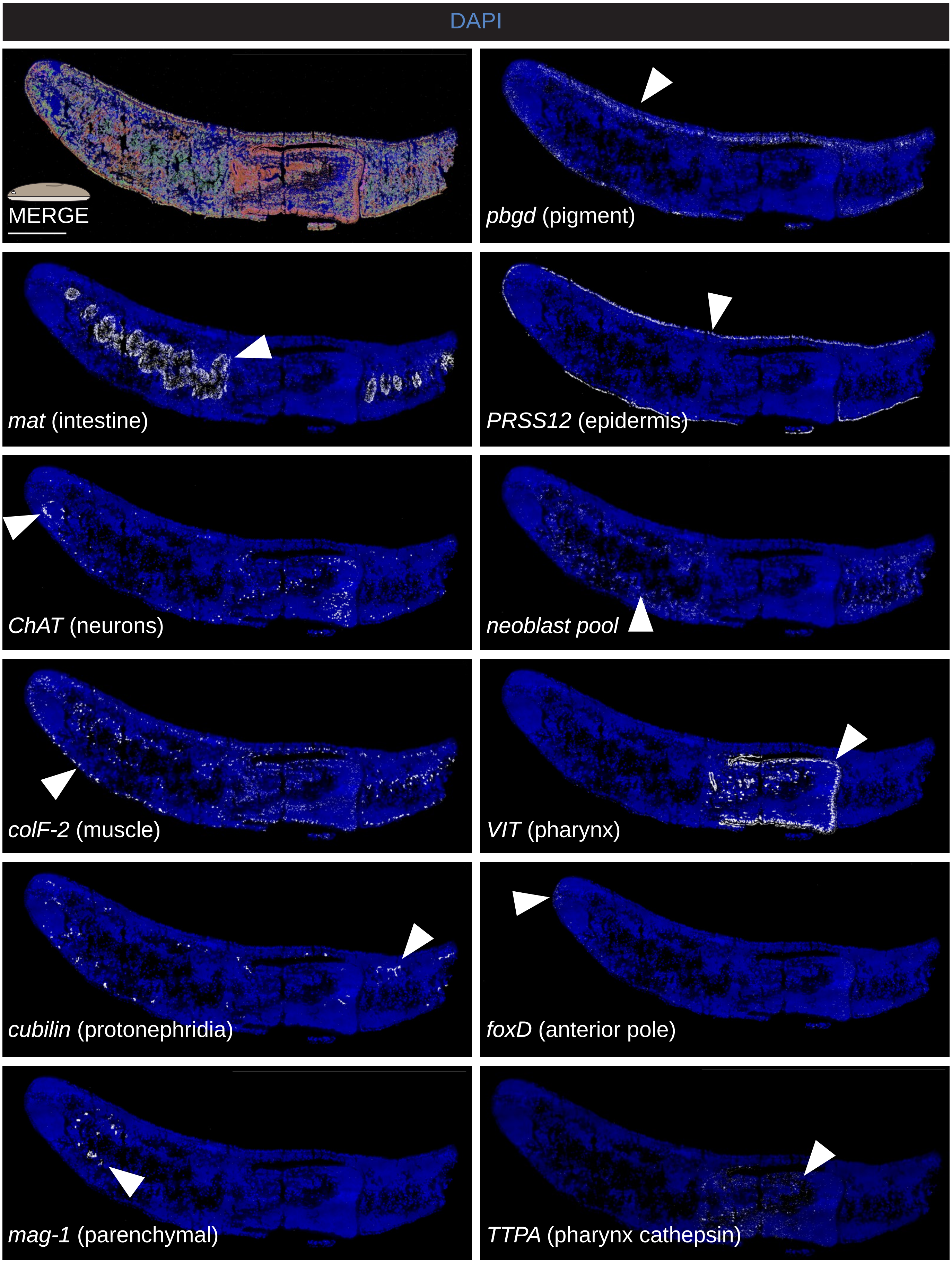
DAPI
MERGE
pbgd (pigment)
mat (intestine)
PRSS12 (epidermis)
ChAT (neurons)
neoblast pool
colF-2 (muscle)
VIT (pharynx)
cubilin (protonephridia)
foxD (anterior pole)
mag-1 (parenchymal)
TTPA (pharynx cathepsin)

Sup Fig. 1

Supplementary Figure 1. MERFISH labels cell types in planarian tissue sections.
MERFISH detection of multiple differentiated cell types in a thin sagittal tissue section. Neoblast pool includes the 13 genes shown in Fig S2. Arrows indicate examples of detected signal. Note: *TTPA* marks a pharynx specific *cathepsin+* cell population. Scale bars, 250 µm. Images shown contain the same region shown in Figure 1.

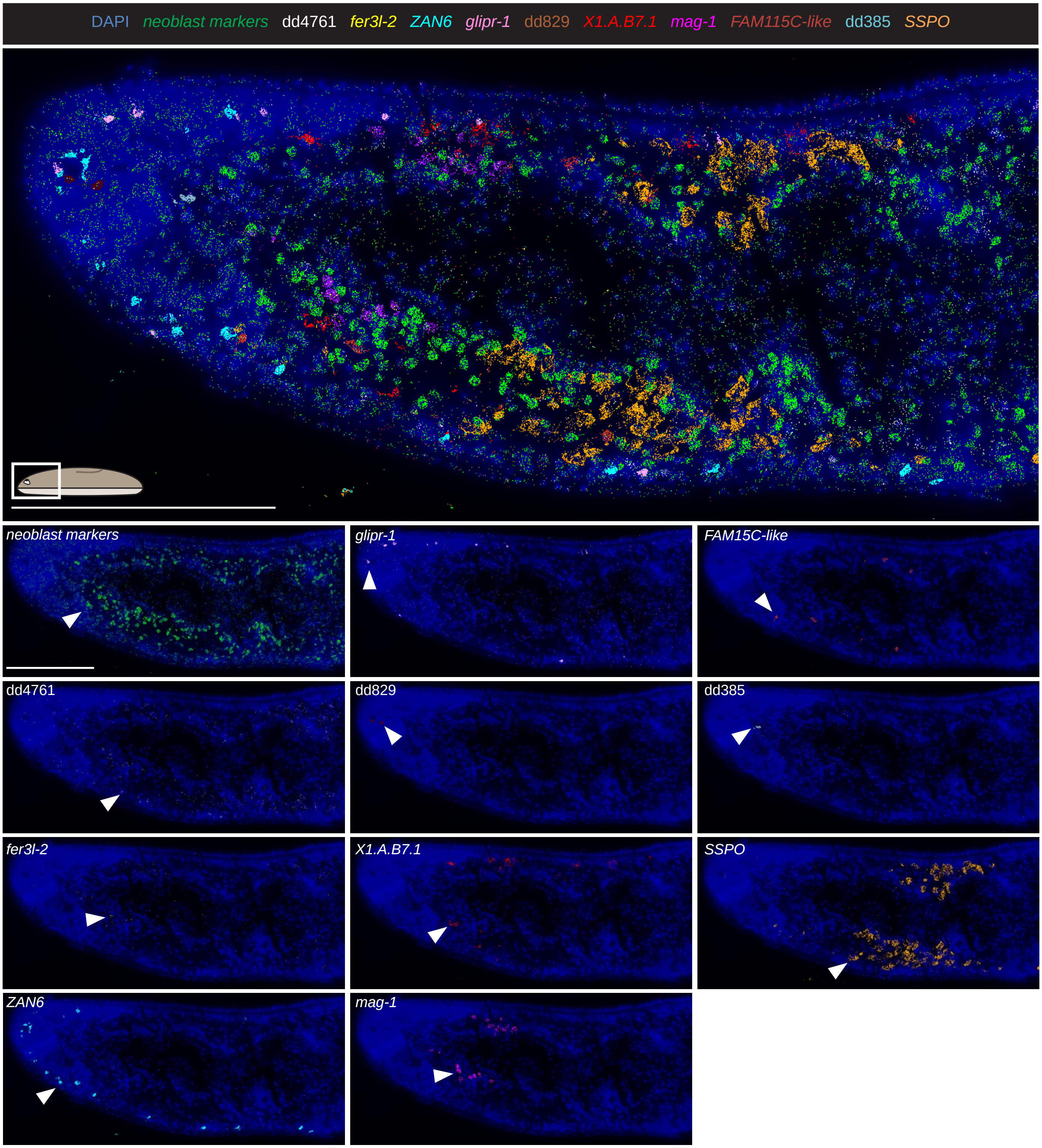
DAPI neoblast markers dd4761 fer3l-2 ZAN6 glipr-1 dd829 X1.A.B7.1 mag-1 FAM115C-like dd385 SSPO
neoblast markers
glipr-1
FAM15C-like
dd4761
dd829
dd385
fer3l-2
X1.A.B7.1
SSPO
ZAN6
mag-1

Sup Fig. 2

Supplementary Figure 2. Diverse parenchymal cell subtypes can be distinguished in single samples by MERFISH

Sagittal section from a planarian head region shows several parenchymal cell subtypes that can be spatially distinguished using MERFISH. Lower panels show each parenchymal cell type individually. Above panel shows merged. Images shown are from the same region as in Figure 1A. Arrows indicate examples of detected signal. Scale bars, 250 µm.

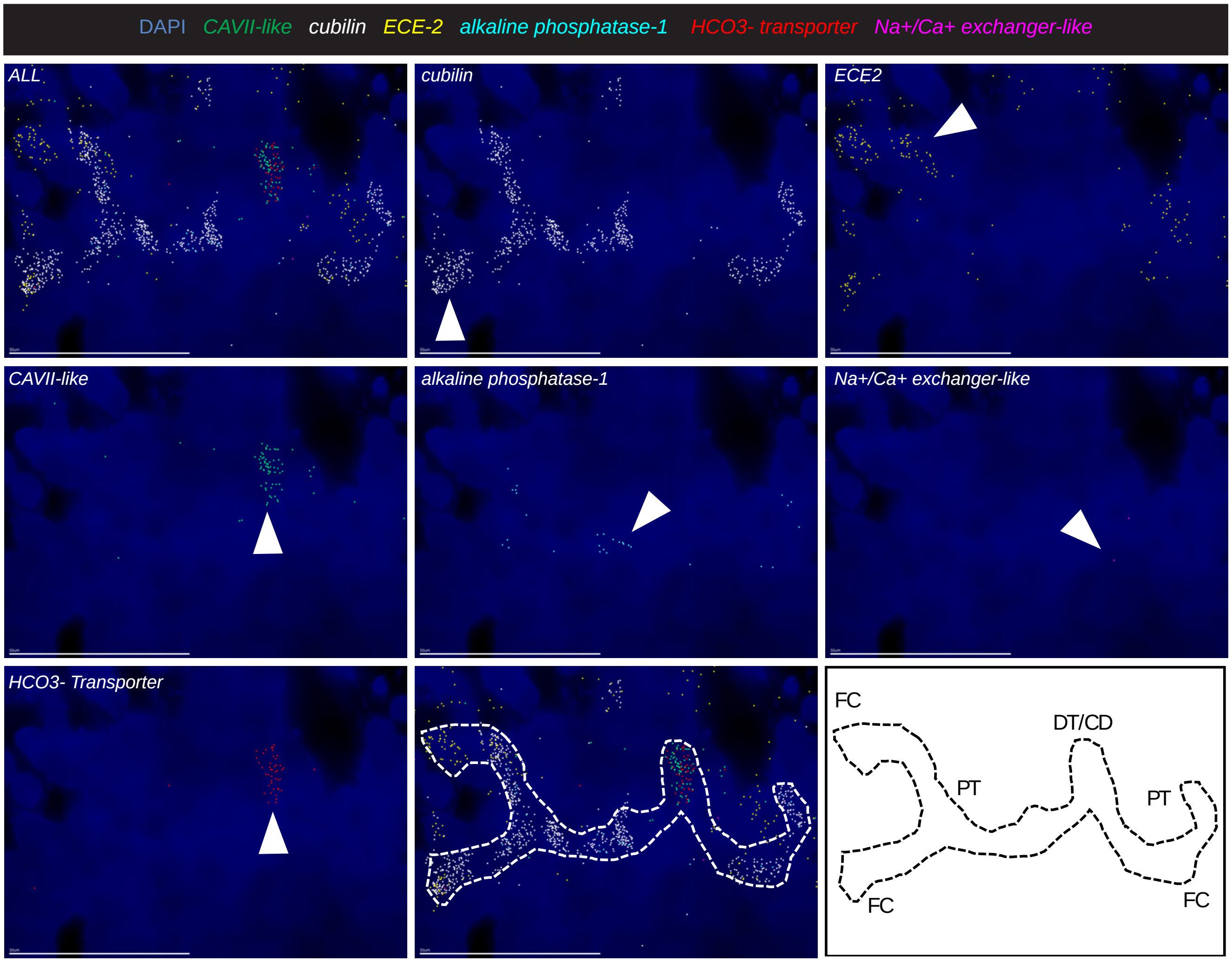
DAPI CAVII-like cubilin ECE-2 alkaline phosphatase-1 HCO3- transporter Na+/Ca+ exchanger-like
ALL
cubilin
ECE2
CAVII-like
alkaline phosphatase-1
Na+/Ca+ exchanger-like
HCO3- Transporter
FC
DT/CD
PT
PT
FC
FC

Sup Fig. 3

Supplementary Figure 3. MERFISH delineates the structure and cell type composition of the protonephridia organ system in single sections

Sagittal planarian section showing overall structure of protonephridia organ system using MERFISH probes for different protonephridia subtypes. Top left image shows pooled gene expression. Bottom middle and right images outline cross section of protonephridia organ system. Arrows indicate examples of detected signal. FC, flame cell; PT, proximal tubule; DT, distal tubule; CD, collecting duct. Scale bars, 50 µm.

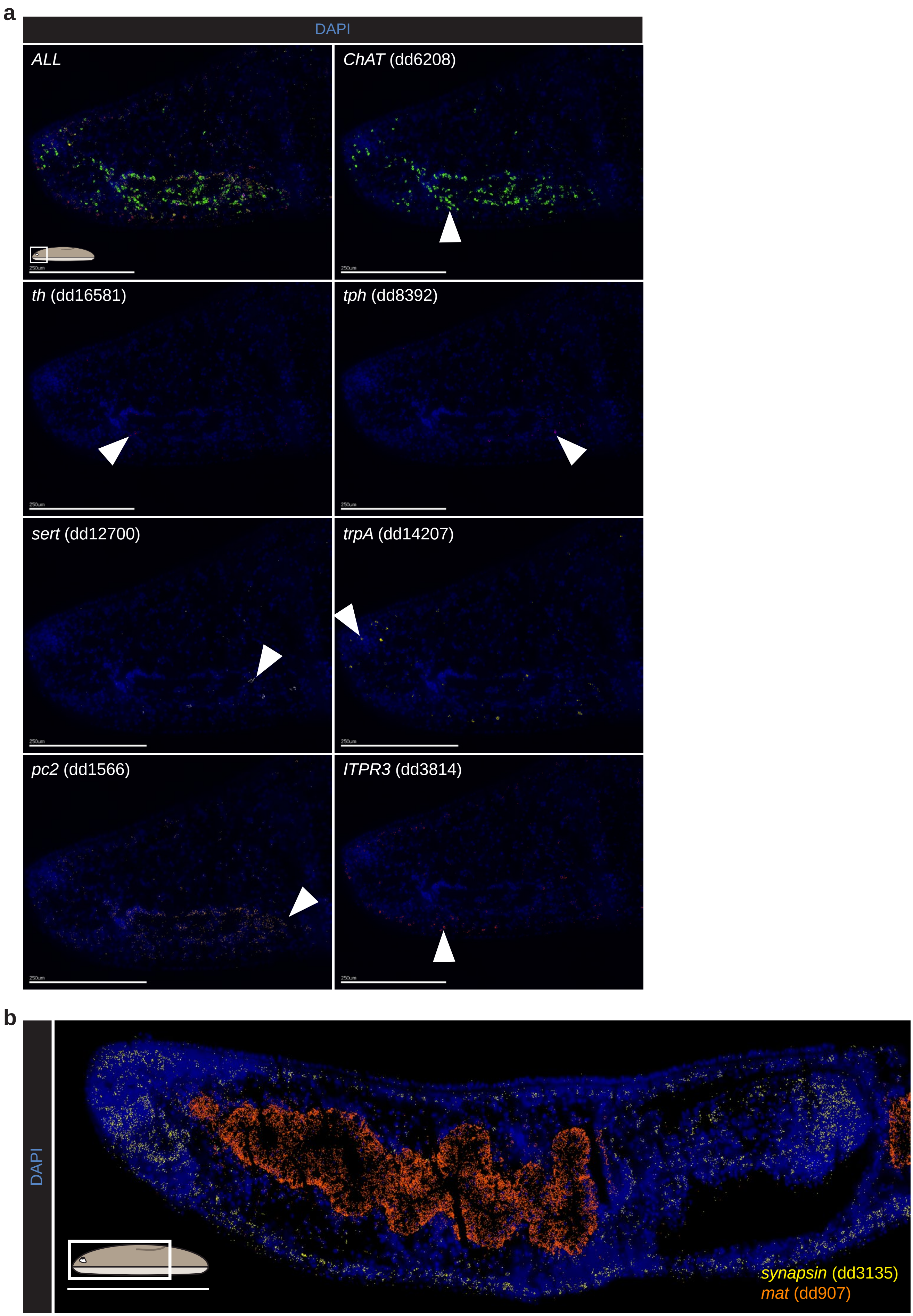
a
DAPI
ALL
ChAT (dd6208)
th (dd16581)
tph (dd8392)
sert (dd12700)
trpA (dd14207)
pc2 (dd1566)
ITPR3 (dd3814)
b
DAPI
synapsin (dd3135)
mat (dd907)

Sup Fig. 4

Supplementary Figure 4. MERFISH labels multiple cell types of the planarian nervous system.

(a) Sagittal section from planarian head depicting multiple neural cell types detected by MERFISH. This includes broad neural markers (e.g., *ChAT*, *pc2*) and subtype specific markers/cells, such as dopaminergic neurons (*th*, *tyrosine hydroxylase*), serotonergic neurons (*sert*), and other populations. Arrows indicate examples of detected signal. Scale bars, 250 µm.
(b) Sagittal section depicting *synapsin* (pan-neural) and *mat* (intestine). (b) includes the same region as contained in Fig 1c. Scale bars, 250 µm.

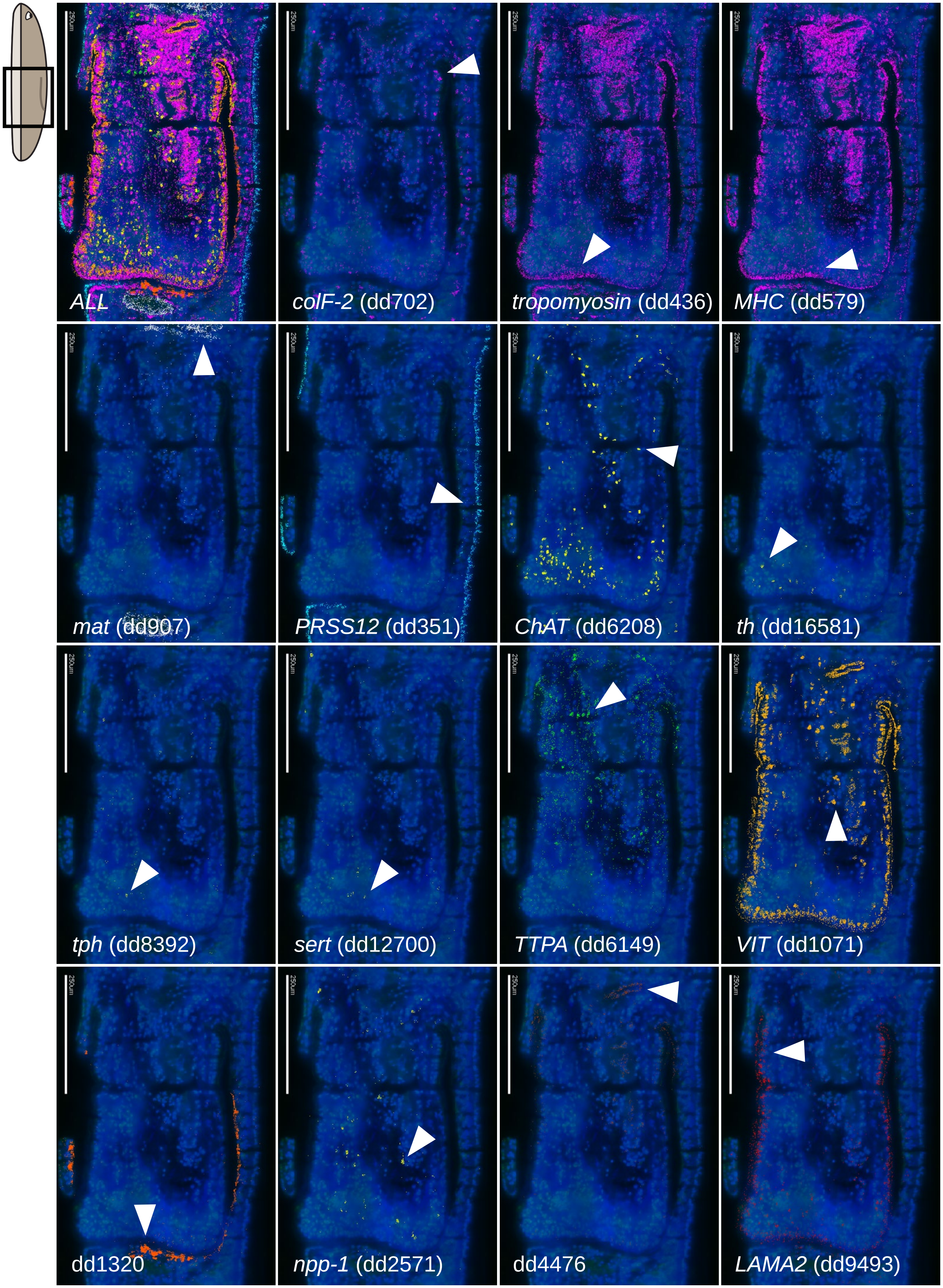
250um
ALL
colF-2 (dd702)
tropomyosin (dd436)
MHC (dd579)
mat (dd907)
PRSS12 (dd351)
ChAT (dd6208)
th (dd16581)
tph (dd8392)
sert (dd12700)
TTPA (dd6149)
VIT (dd1071)
dd1320
npp-1 (dd2571)
dd4476
LAMA2 (dd9493)

Sup Fig. 5

Supplementary Figure 5. Pharynx anatomy and cell-type diversity labeled in single samples by MERFISH

Sagittal section depicting the detection of multiple different cell types of the planarian pharynx (a muscular feeding tube located in the mid-body of the animal) using MERFISH. Marker genes represent diverse cell types within the pharynx such as muscle (e.g., *colF-2, tropomyosin, MHC*), neurons (e.g., *ChAT, th, sert*), a pharynx specific *cathepsin+* cell population (*TTPA),* epithelial cells lining the pharynx (e.g., dd1320), epithelial cells lining the pharyngeal cavity (e.g., *LAMA2*), and cells connecting the pharynx to the planarian body (e.g., dd4476). The intestine (*mat*) and epidermis (*PRSS12*) outside of the pharynx is robustly detected by MERFISH. Arrows indicate examples of detected signal. Scale bars, 250 µm.

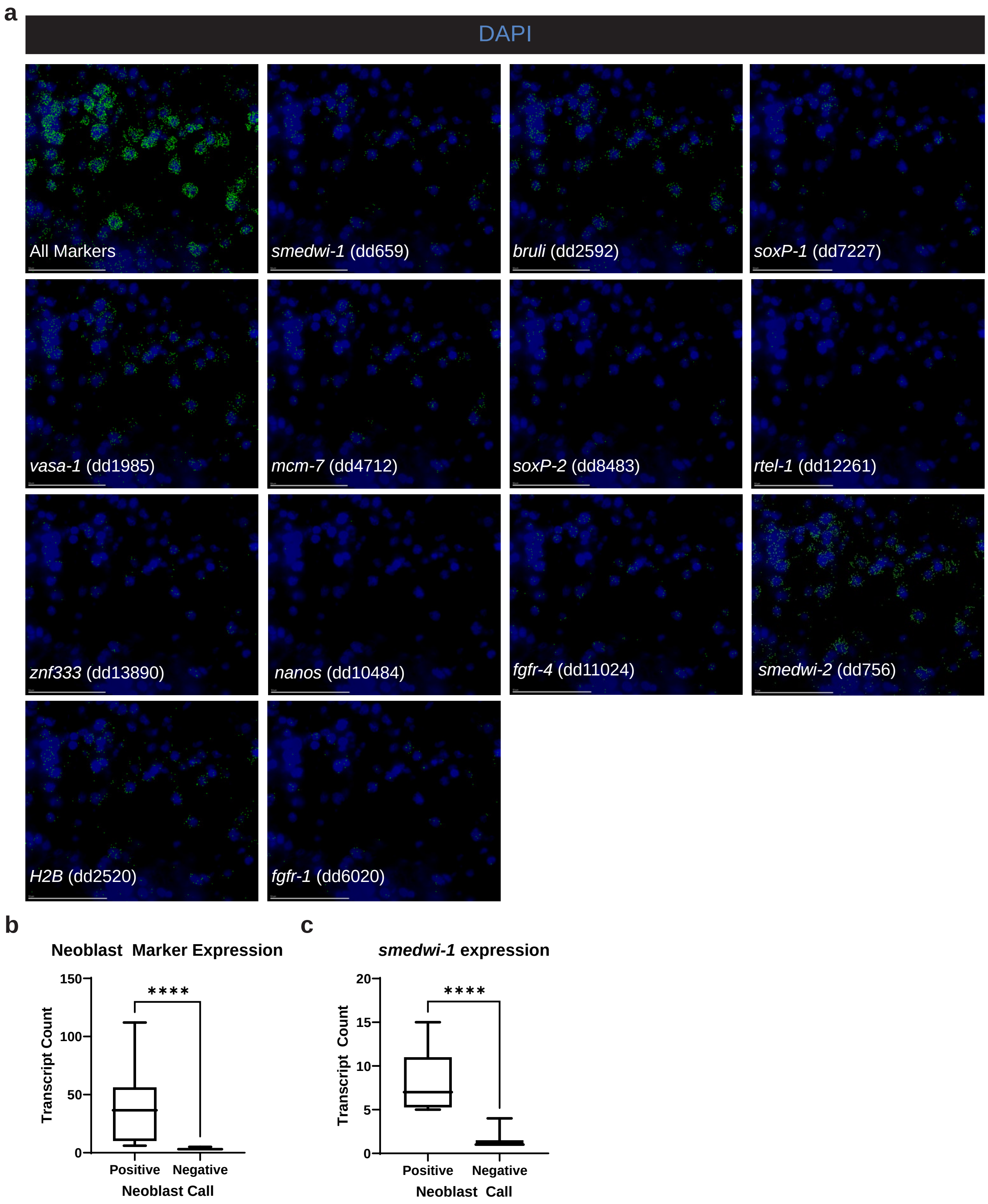
a
DAPI
All Markers
smedwi-1 (dd659)
bruli (dd2592)
soxP-1 (dd7227)
vasa-1 (dd1985)
mcm-7 (dd4712)
soxP-2 (dd8483)
rtel-1 (dd12261)
znf333 (dd13890)
nanos (dd10484)
fgfr-4 (dd11024)
smedwi-2 (dd756)
H2B (dd2520)
fgfr-1 (dd6020)
b
Neoblast Marker Expression
Transcript Count
150
100
50
0
****
Positive
Negative
Neoblast Call
c
smedwi-1 expression
Transcript Count
20
15
10
5
0
****
Positive
Negative
Neoblast Call

Sup Fig. 6

Supplementary Figure 6. Pooled gene expression is used to detect cell types
(a) Neoblasts are identified by the pooled expression of neoblast-specific and neoblast-enriched genes. MERFISH probes for 13 total neoblasts genes were used. This 13-gene pool is used for all neoblast pool MERFISH experiments noted in the study. Scale bars, 50 µm. (b) Quantification of neoblast marker expression in called neoblasts utilized for analysis by MERFISH. Two-tailed Welch's t-test, **** = p<0.0001. The upper and lower hinges correspond to 25th and 75th percentiles, whiskers represent the smallest and largest values, and the line corresponds to the median. (c) Quantification of *smedwi-1* expression in called neoblasts. Two-tailed Welch's t-test, **** = p<0.0001. See boxplot definition in (b). N = 54 (Positive), and 46 (Negative) in (b) and N = 56 (Positive) and 53 (Negative) in (c). Source data are provided as a Source Data file.

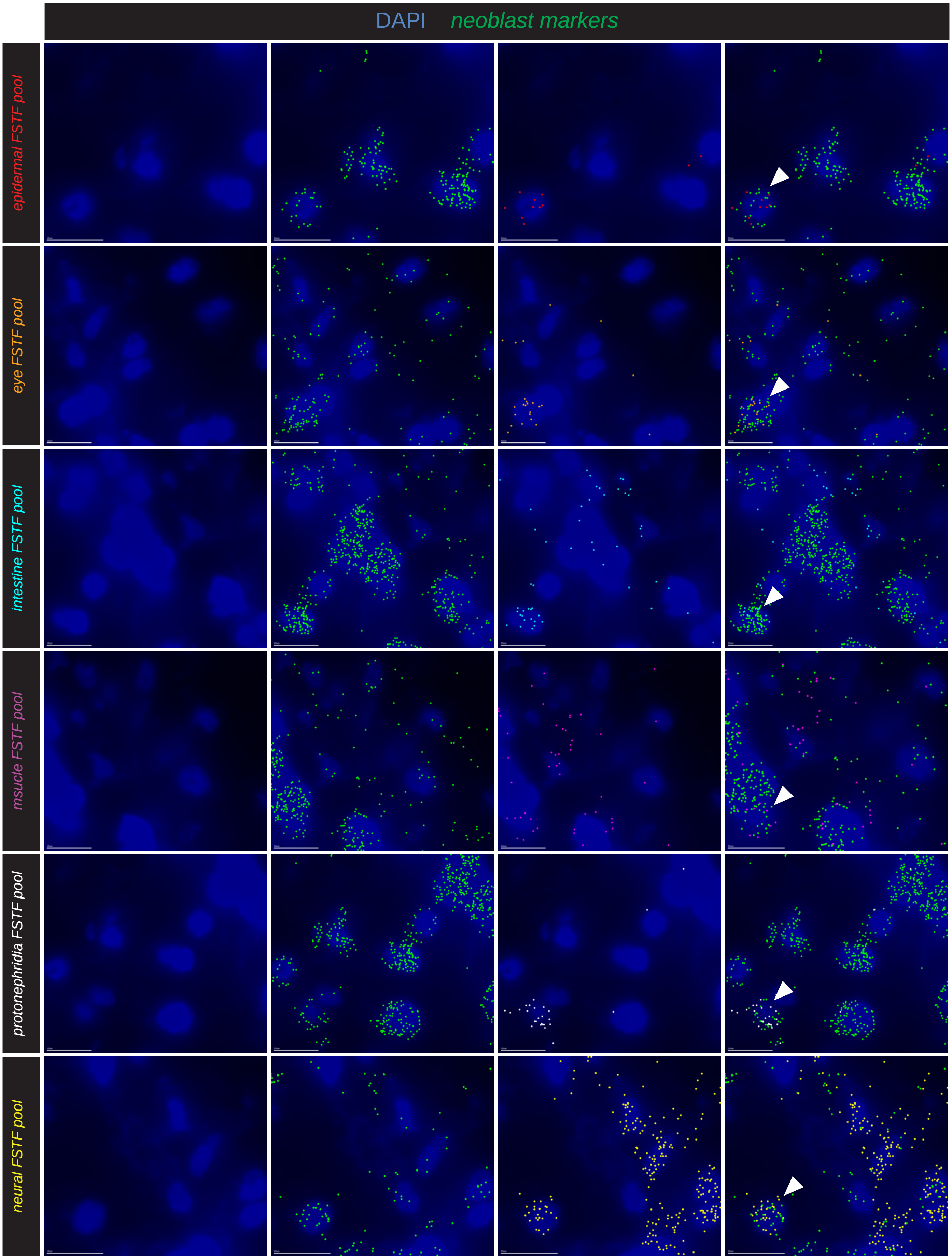

DAPI
neoblast markers
epidermal FSTF pool
eye FSTF pool
intestine FSTF pool
msucle FSTF pool
protonephridia FSTF pool
neural FSTF pool

Sup Fig. 7

Supplementary Figure 7. Specialized neoblasts are labeled by FSTF expression
Specialized neoblasts within ventral anterior regions of planarians are labeled by the pooled expression of neoblast markers and the pooled expression of multiple tissue-enriched FSTFs. Each row shows labeling of one class of specialized neoblasts. Middle left columns show neoblast pool alone. Middle right columns show tissue-enriched FSTF pool alone. Right columns show merged images. White arrows show FSTF-positive cell. Genes for tissue-specific FSTF pools are listed in Supplementary Table 2. Scale bars, 10 µm.

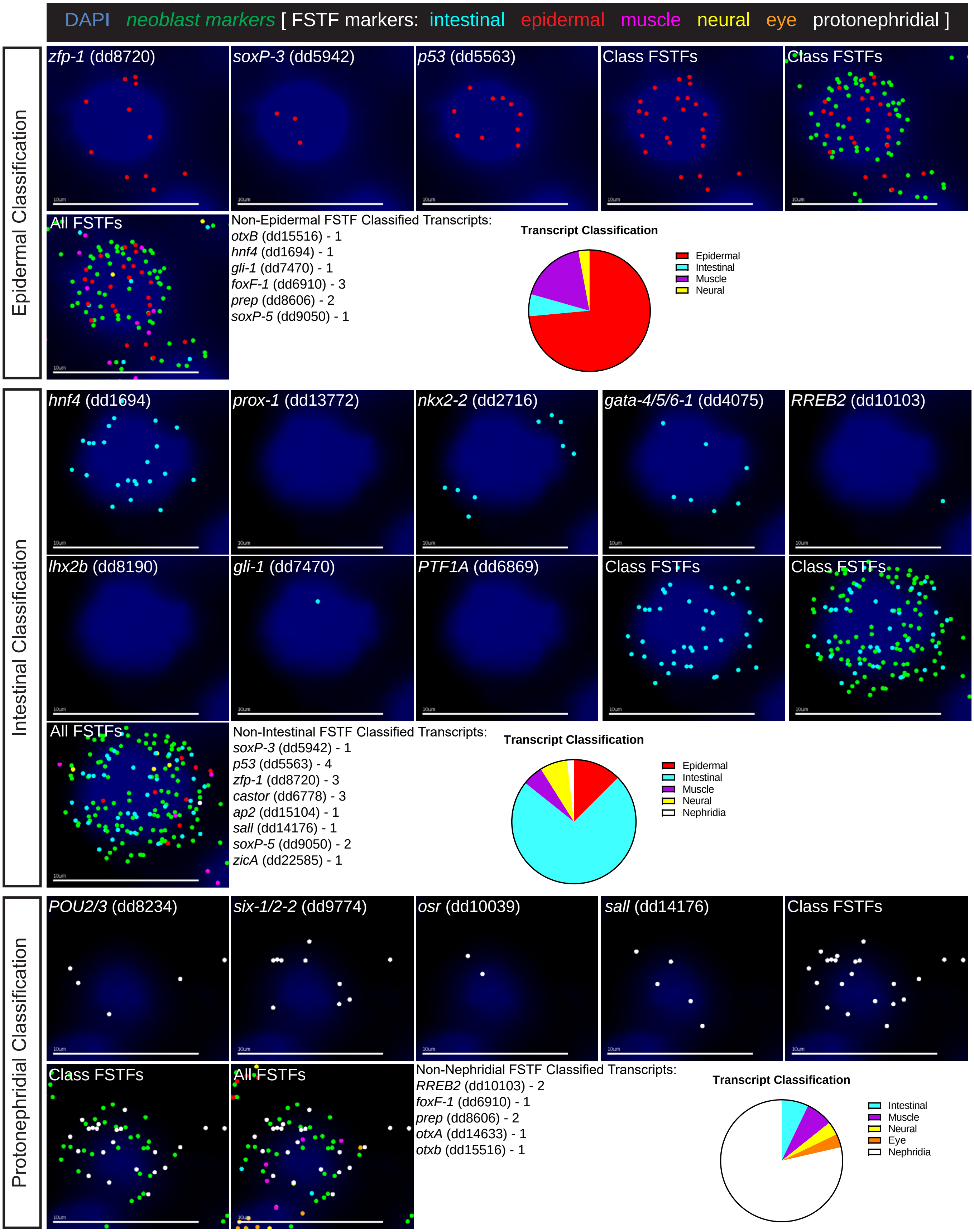
DAPI neoblast markers [FSTF markers: intestinal epidermal muscle neural eye protonephridial]
Epidermal Classification
zfp-1 (dd8720)
soxP-3 (dd5942)
p53 (dd5563)
Class FSTFs
Class FSTFs
All FSTFs
Non-Epidermal FSTF Classified Transcripts:
otxB (dd15516) - 1
hnf4 (dd1694) - 1
gli-1 (dd7470) - 1
foxF-1 (dd6910) - 3
prep (dd8606) - 2
soxP-5 (dd9050) - 1
Transcript Classification
Epidermal
Intestinal
Muscle
Neural
Intestinal Classification
hnf4 (dd1694)
prox-1 (dd13772)
nkx2-2 (dd2716)
gata-4/5/6-1 (dd4075)
RREB2 (dd10103)
lhx2b (dd8190)
gli-1 (dd7470)
PTF1A (dd6869)
Class FSTFs
Class FSTFs
All FSTFs
Non-Intestinal FSTF Classified Transcripts:
soxP-3 (dd5942) - 1
p53 (dd5563) - 4
zfp-1 (dd8720) - 3
castor (dd6778) - 3
ap2 (dd15104) - 1
sall (dd14176) - 1
soxP-5 (dd9050) - 2
zicA (dd22585) - 1
Transcript Classification
Epidermal
Intestinal
Muscle
Neural
Nephridia
Protonephridial Classification
POU2/3 (dd8234)
six-1/2-2 (dd9774)
osr (dd10039)
sall (dd14176)
Class FSTFs
Class FSTFs
All FSTFs
Non-Nephridial FSTF Classified Transcripts:
RREB2 (dd10103) - 2
foxF-1 (dd6910) - 1
prep (dd8606) - 2
otxA (dd14633) - 1
otxb (dd15516) - 1
Transcript Classification
Intestinal
Muscle
Neural
Eye
Nephridia

Sup Fig. 8

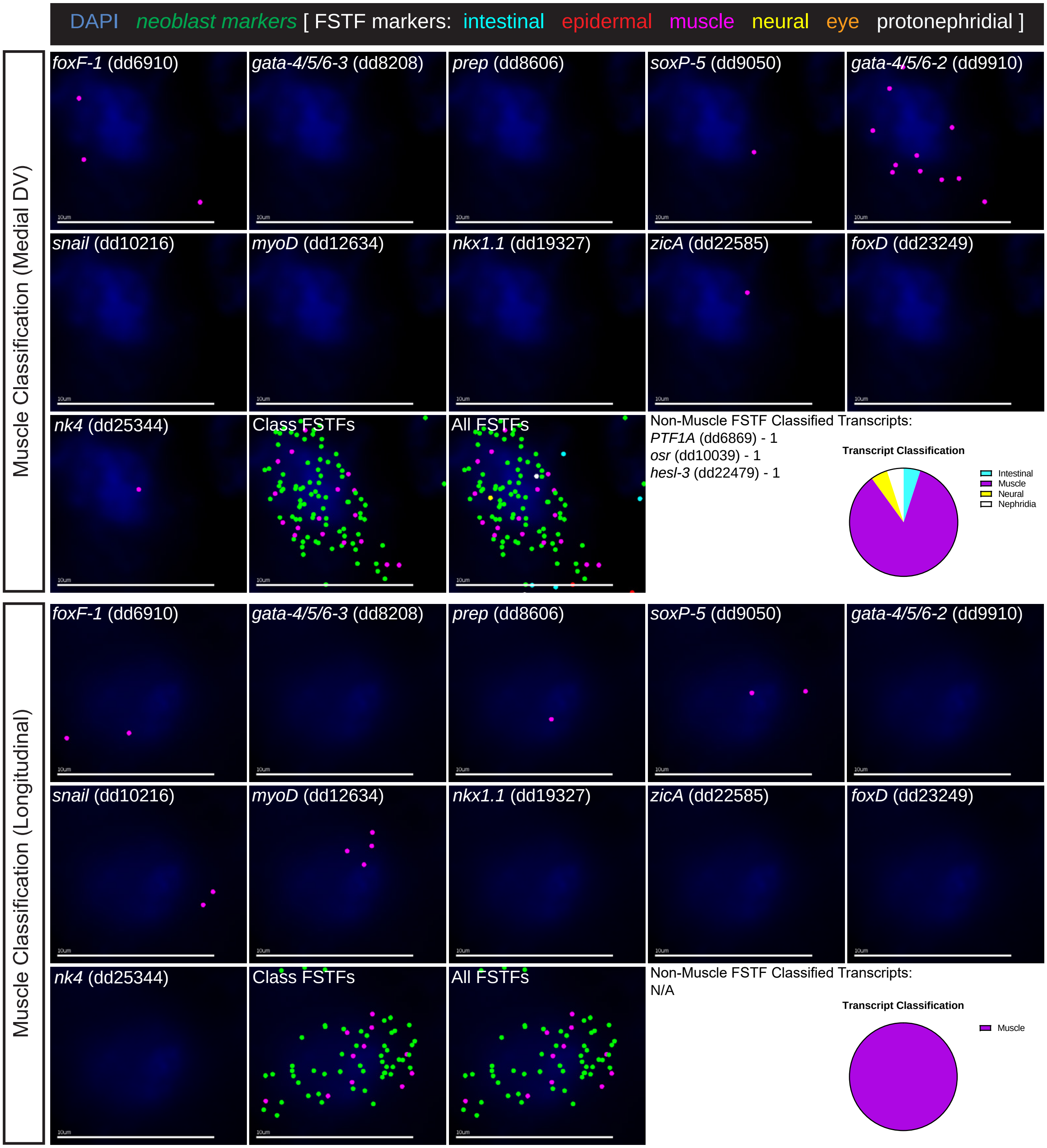
DAPI neoblast markers [FSTF markers: intestinal epidermal muscle neural eye protonephridial]
Muscle Classification (Medial DV)
foxF-1 (dd6910)
gata-4/5/6-3 (dd8208)
prep (dd8606)
soxP-5 (dd9050)
gata-4/5/6-2 (dd9910)
snail (dd10216)
myoD (dd12634)
nkx1.1 (dd19327)
zicA (dd22585)
foxD (dd23249)
nk4 (dd25344)
Class FSTFs
All FSTFs
Non-Muscle FSTF Classified Transcripts:
PTF1A (dd6869) - 1
osr (dd10039) - 1
hesl-3 (dd22479) - 1
Transcript Classification
Intestinal
Muscle
Neural
Nephridia
Muscle Classification (Longitudinal)
foxF-1 (dd6910)
gata-4/5/6-3 (dd8208)
prep (dd8606)
soxP-5 (dd9050)
gata-4/5/6-2 (dd9910)
snail (dd10216)
myoD (dd12634)
nkx1.1 (dd19327)
zicA (dd22585)
foxD (dd23249)
nk4 (dd25344)
Class FSTFs
All FSTFs
Non-Muscle FSTF Classified Transcripts:
N/A
Transcript Classification
Muscle

Sup Fig. 9

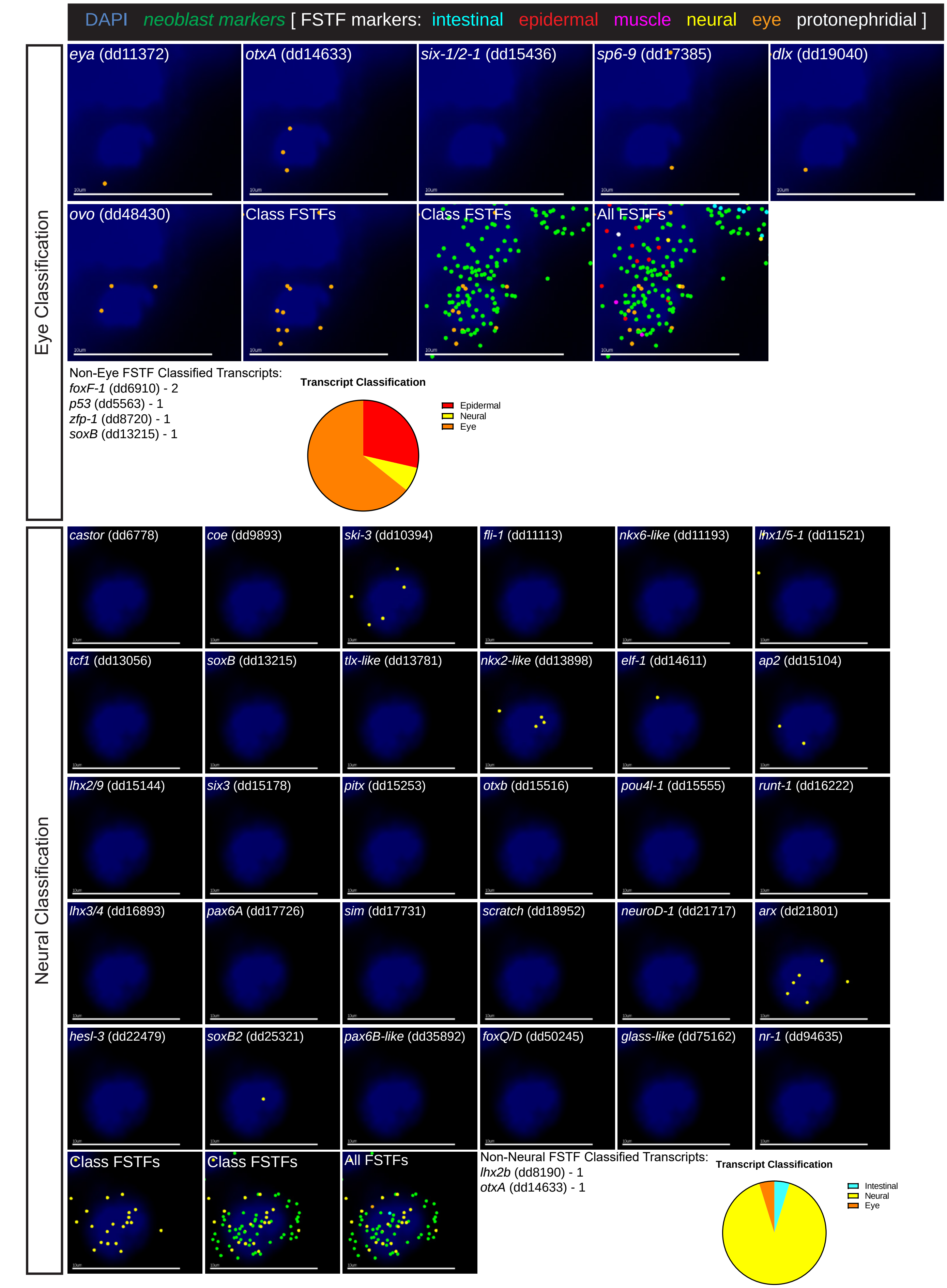
DAPI neoblast markers [FSTF markers: intestinal epidermal muscle neural eye protonephridial]
Eye Classification
eya (dd11372)
otxA (dd14633)
six-1/2-1 (dd15436)
sp6-9 (dd17385)
dlx (dd19040)
ovo (dd48430)
Class FSTFs
Class FSTFs
All FSTFs
Non-Eye FSTF Classified Transcripts:
foxF-1 (dd6910) - 2
p53 (dd5563) - 1
zfp-1 (dd8720) - 1
soxB (dd13215) - 1
Transcript Classification
Epidermal
Neural
Eye
Neural Classification
castor (dd6778)
coe (dd9893)
ski-3 (dd10394)
fli-1 (dd11113)
nkx6-like (dd11193)
lhx1/5-1 (dd11521)
tcf1 (dd13056)
soxB (dd13215)
tlx-like (dd13781)
nkx2-like (dd13898)
elf-1 (dd14611)
ap2 (dd15104)
lhx2/9 (dd15144)
six3 (dd15178)
pitx (dd15253)
otxb (dd15516)
pou4l-1 (dd15555)
runt-1 (dd16222)
lhx3/4 (dd16893)
pax6A (dd17726)
sim (dd17731)
scratch (dd18952)
neuroD-1 (dd21717)
arx (dd21801)
hesl-3 (dd22479)
soxB2 (dd25321)
pax6B-like (dd35892)
foxQ/D (dd50245)
glass-like (dd75162)
nr-1 (dd94635)
Class FSTFs
Class FSTFs
All FSTFs
Non-Neural FSTF Classified Transcripts:
lhx2b (dd8190) - 1
otxA (dd14633) - 1
Transcript Classification
Intestinal
Neural
Eye

Sup Fig. 10

Supplementary Figures 8-10. Pooled expression of multiple FSTFs (FSTF signatures) is used to label specialized neoblasts

Images showing how pooled expression of multiple FSTF transcripts is used to create a signature that identifies a specialized neoblast of a given class. Neoblasts are from ventral anterior regions of the animal. Classification of seven specialized neoblast class are shown (Fig. S8: epidermis, intestine, protonephridia; Fig. S9: medial DV (dorsoventral) muscle, longitudinal muscle; Fig. S10: eye and neural). Images show the expression of individual transcripts, along with pooled expression. For each specialized neoblast type, the total number of non-class FSTF transcripts found in the cell were also calculated. Pie graphs depict and classify the proportion of different FSTF transcripts found in the cells imaged. Pie graphs indicate that although specialized neoblasts most abundantly express transcripts of one class (e.g., epidermal pool, intestine pool), transcripts for other classes can be found in the same neoblasts as well.

Scale bars, 10 µm. Source data are provided as a Source Data file.

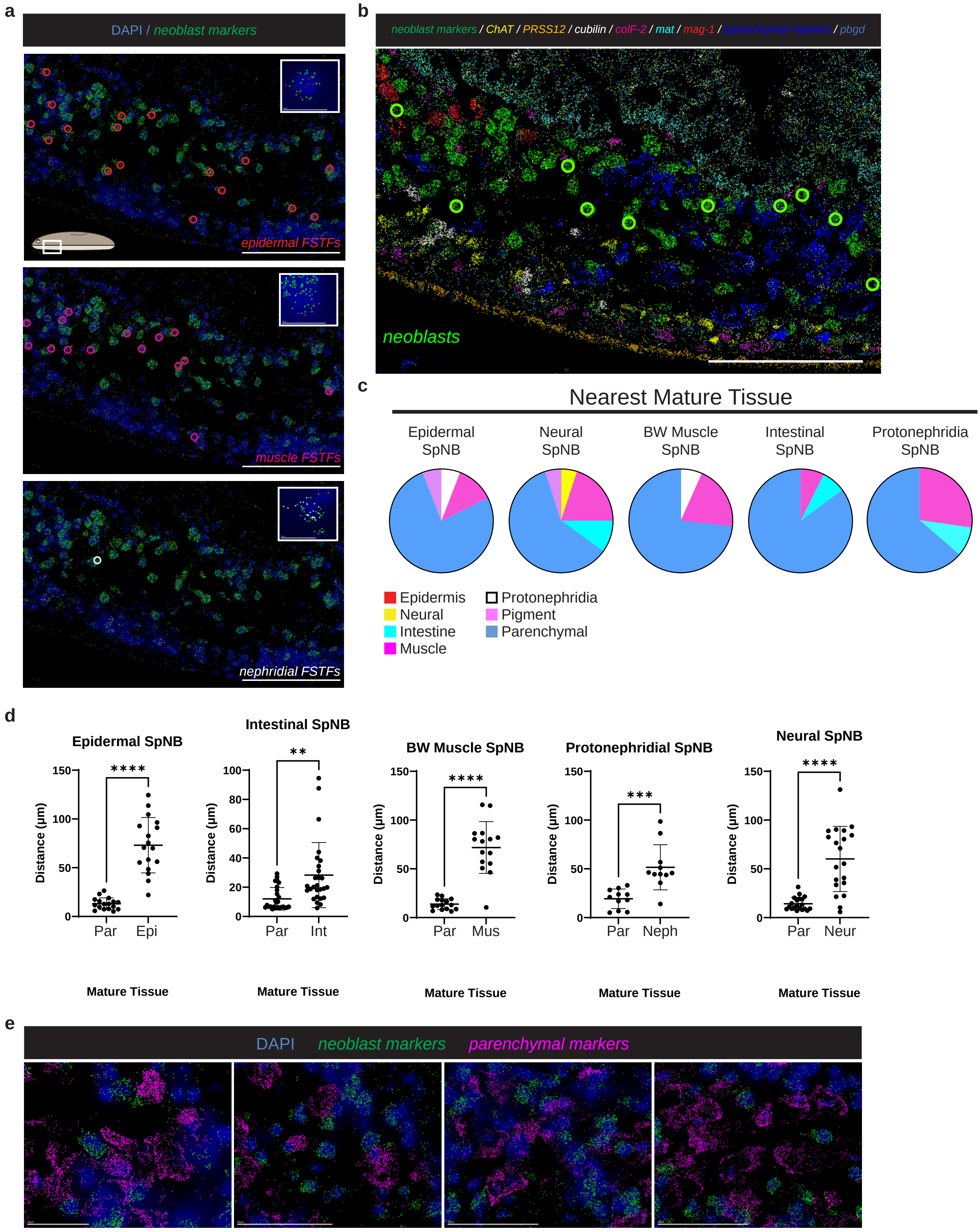
a
DAPI / neoblast markers
epidermal FSTFs
muscle FSTFs
nephridial FSTFs
b
neoblast markers / ChAT / PRSS12 / cubilin / colF-2 / mat / mag-1 / parenchymal markers / pbgd
neoblasts
c
Nearest Mature Tissue
Epidermal SpNB
Neural SpNB
BW Muscle SpNB
Intestinal SpNB
Protonephridia SpNB
Epidermis
Neural
Intestine
Muscle
Protonephridia
Pigment
Parenchymal
d
Epidermal SpNB
Intestinal SpNB
BW Muscle SpNB
Protonephridial SpNB
Neural SpNB
Distance (μm)
****
**
****
***
****
Par
Epi
Int
Mus
Neph
Neur
Mature Tissue
e
DAPI neoblast markers parenchymal markers

Sup Fig. 11

Supplementary Figure 11. Spatial mapping of specialized neoblasts and their mature target tissues in intact animal sections using MERFISH.

(a) Sagittal section from ventral side of animal using MERFISH. Multiple specialized neoblast classes are distributed throughout the parenchyma of the animal. Colored circles label a given specialized neoblast (SpNB) class. Insets show representative SpNB. Images depict the same region as Figure 1. Scale bar, 100 µm. (b) Same sagittal section as (a), showing all neoblasts distributed within the parenchyma. Green circles represent neoblasts that were not assigned to a fate-specialized class. Scale bar, 100 µm. Data from cells queried in Fig. 1c. (c) The proportion of the nearest mature tissues for each identified SpNB from Figure 1B and S11A. Colors represent different mature tissues. BW, body-wall. (d) Distance measurements of specialized neoblasts to parenchymal mature tissues or target mature tissues. Two-sided Welch's t-test, **** = $p<0.0001$, *** = $p=0.0009$, ** = $p=0.0011$. Data from cells queried in Fig. 1c. The central line represents the mean and error bars represent standard deviation. Par, parenchymal; Epi, epidermal; Int, intestinal; Mus, body-wall muscle; Neph, protonephridial; Neur, neural. (e) Neoblasts are located in close proximity to differentiated parenchymal cell types. Scale bars, 50 µm. Source data are provided as a Source Data file.

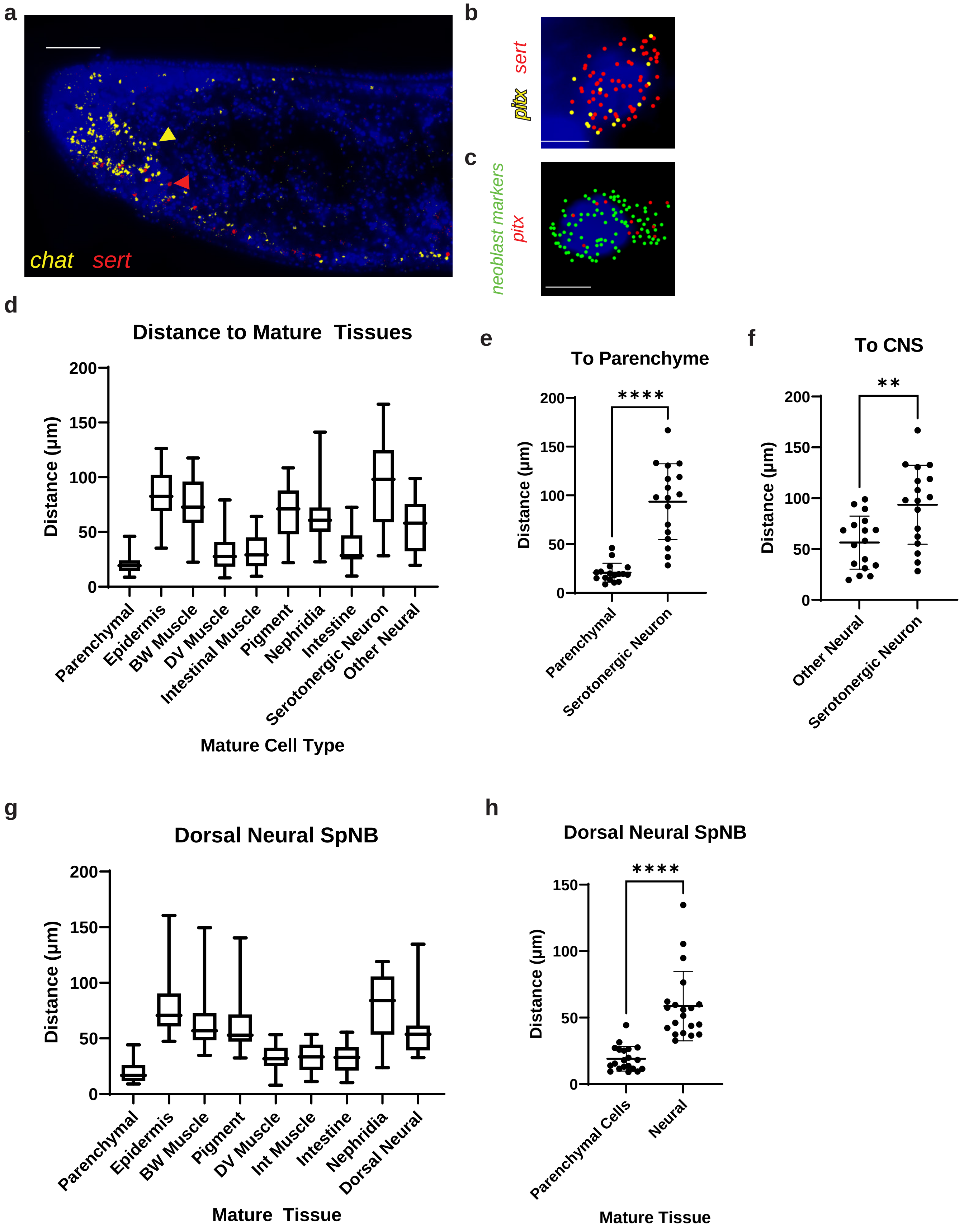

a
chat sert
b
pitx sert
c
neoblast markers pitx
d
Distance to Mature Tissues
Distance (µm)
Parenchymal
Epidermis
BW Muscle
DV Muscle
Intestinal Muscle
Pigment
Nephridia
Intestine
Serotonergic Neuron
Other Neural
Mature Cell Type
e
To Parenchyme
****
Distance (µm)
Parenchymal
Serotonergic Neuron
f
To CNS
**
Distance (µm)
Other Neural
Serotonergic Neuron
g
Dorsal Neural SpNB
Distance (µm)
Parenchymal
Epidermis
BW Muscle
Pigment
DV Muscle
Int Muscle
Intestine
Nephridia
Dorsal Neural
Mature Tissue
h
Dorsal Neural SpNB
****
Distance (µm)
Parenchymal Cells
Neural
Mature Tissue

Sup Fig. 12

Supplementary Figure 12. Spatial mapping of neural specialized neoblasts to mature tissues.
(a) MERFISH images of cholinergic neurons (yellow arrow) and serotonergic neurons (red arrow) in the anterior of a planarian tissue section. Scale bar, 100 µm. Region depicts same region as in Fig. 1c (b) Expression of *pitx* in mature serotonergic neurons (*sert*+). Scale bar, 5 µm. (c) A *pitx*+ serotonergic neural specialized neoblast. Scale bar, 5 µm. (d) Distance measurements of serotonergic neural specialized neoblasts to mature tissues. N = 17. (e) Distance measurements of serotonergic neural specialized neoblasts from (d) to mature parenchymal cell types or mature serotonergic neurons. (f) Distance measurements of serotonergic neural specialized neoblasts from (d) to mature serotonergic or non-serotonergic neurons. (g) Distance measurement of dorsal neural specialized neoblasts to dorsal peripheral neurons. N = 20. (h) Distance measurements of dorsal neural specialized neoblasts from (g) to mature parenchymal or neural cell types. Two-sided Welch's t-test in (e), (f), and (h). The bounds of boxes in (d) and (g) correspond to 25th and 75th percentiles, whiskers represent the smallest and largest values, and the line corresponds to the median. Data in (e), (f), and (g) presented as mean ± standard deviation. ** = p=0.0028, **** = p<0.0001. Source data are provided as a Source Data file.

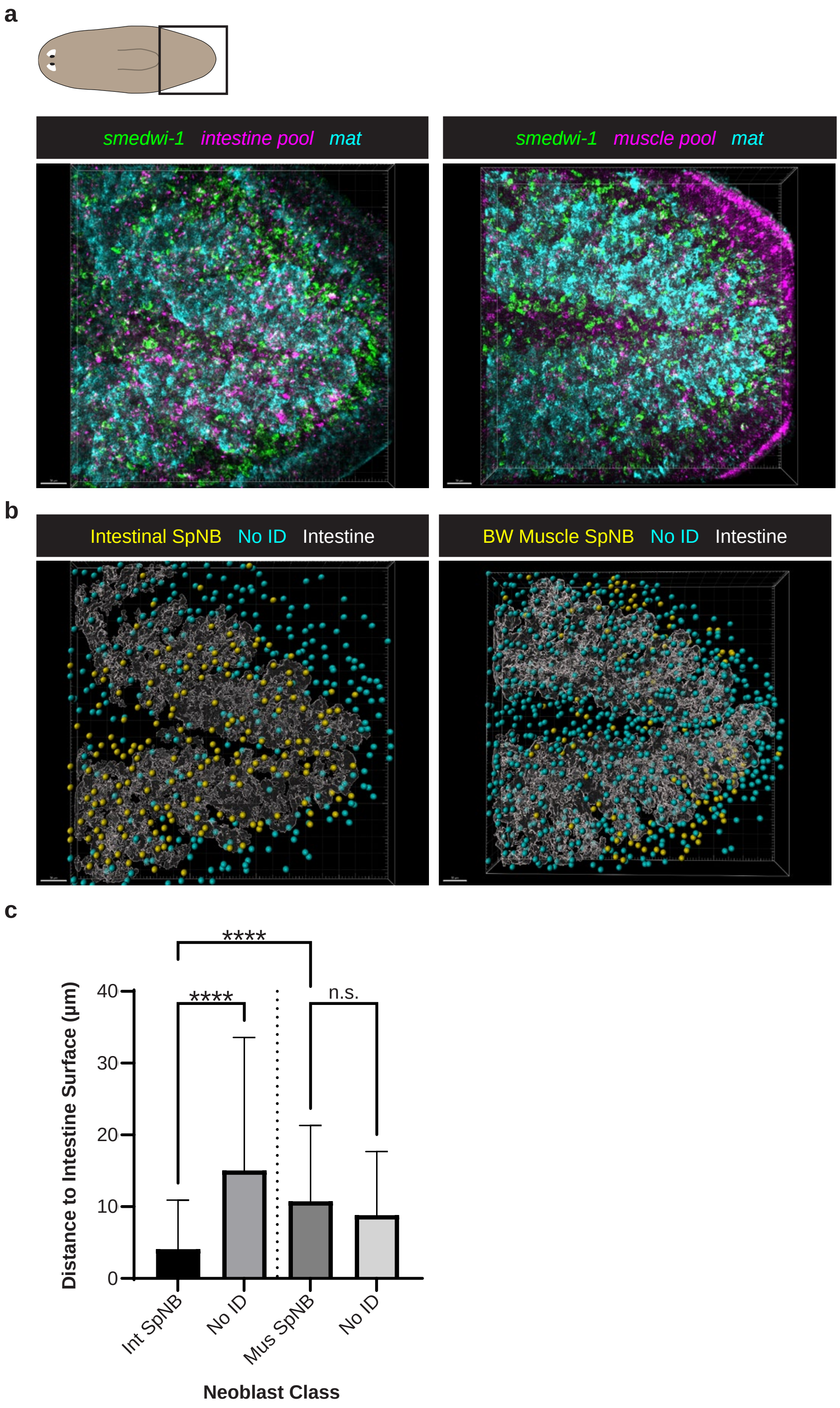
a
smedwi-1 intestine pool mat
smedwi-1 muscle pool mat
b
Intestinal SpNB No ID Intestine
BW Muscle SpNB No ID Intestine
c
****
****
n.s.
Distance to Intestine Surface (µm)
0
10
20
30
40
Int SpNB
No ID
Mus SpNB
No ID
Neoblast Class

Sup Fig. 13

Supplementary Figure 13. Three-dimensional spatial mapping of specialized neoblasts and target tissues using whole-mount FISH.

(a) FISH images of the planarian tail assessing the spatial location of intestine-specialized neoblasts and muscle-specialized neoblasts relative to the mature intestine. Scale bar, 30 µm. Intestine pool = *gata4/5/6-1* and *hnf-4*. Muscle pool = *myoD* and *snail*. (b) Imaris-generated maps modeling the FISH images shown in (a). Scale bar, 30 µm. (c). Distance measurements of various neoblast classes to intestine surface. Measurements calculated using Imaris surface detection based on maps in (b). N = 171 (Int SpNB), 288 (No ID left), 61 (Mus SpNB), 394 (No ID right). Int SpNB, intestine-specialized neoblast. Mus SpNB, body wall muscle-specialized neoblast. No ID, unlabeled neoblast (non-muscle, non-intestine). Data presented as mean ± standard deviation. Two-sided Welch's t-test, **** = $p<0.0001$, n.s. = not significant. Source data are provided as a Source Data file.

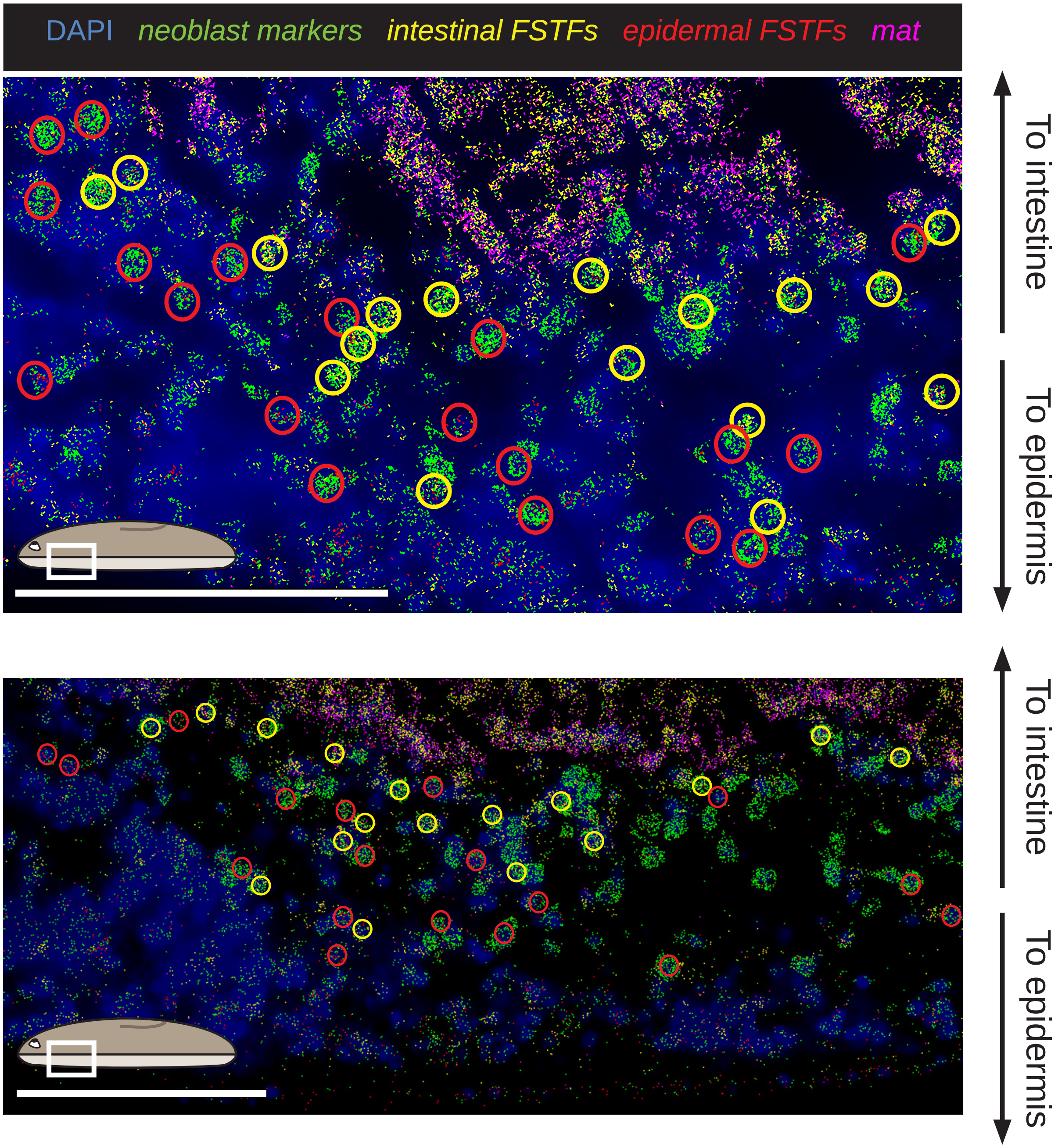
DAPI neoblast markers intestinal FSTFs epidermal FSTFs mat
To intestine
To epidermis
To intestine
To epidermis

Sup Fig. 14

Supplementary Figure 14. Epidermal and intestinal specialized neoblasts are found in broad and overlapping spatial domains.

Intestine and epidermal specialized neoblasts (SpNBs) are present in broad and overlapping spatial domains in ventral, pre-pharyngeal areas of the animal. Images represent regions taken from independent sections. Yellow circles represent detected intestinal SpNBs, red circles represent detected epidermal SpNBs. Scale bars, 100 µm.

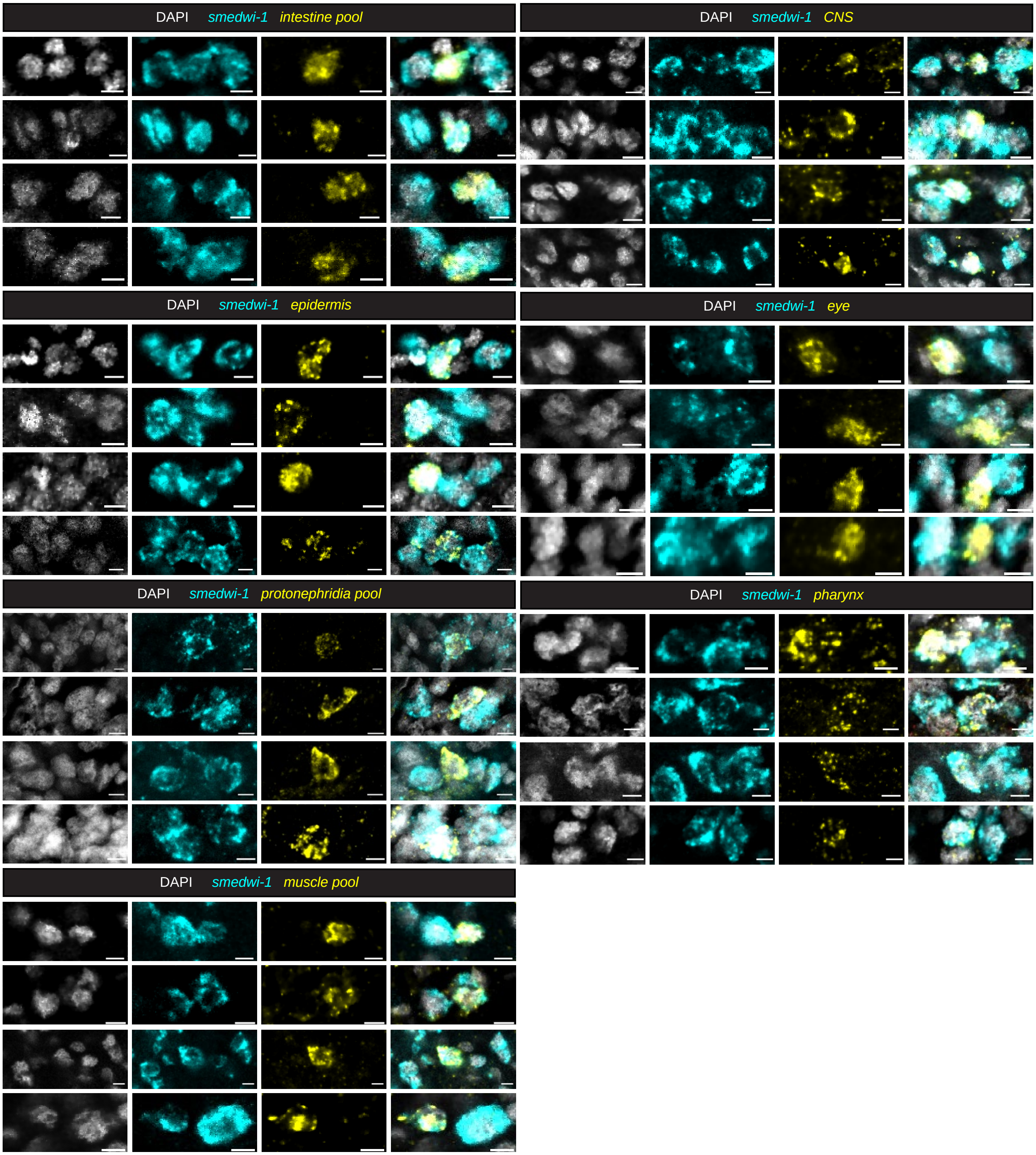
DAPI smedwi-1 intestine pool
DAPI smedwi-1 CNS
DAPI smedwi-1 epidermis
DAPI smedwi-1 eye
DAPI smedwi-1 protonephridia pool
DAPI smedwi-1 pharynx
DAPI smedwi-1 muscle pool

Sup Fig. 15

Supplementary Figure 15. Specialized neoblasts of the same fate do not exist in local clusters.

Whole-mount FISH images showing specialized neoblasts of various classes in close proximity to neoblasts not of their respective class. Images shown are the same as in Figure 2A. Scale bar, 5 µm. Genes for tissue-specific FSTF pools: epidermis (*soxP-3*), intestine (*hnf-4, gata4/5/6-1*), protonephridia (*POU2/3, six-1/2-2*), muscle (*myoD, snail*), CNS (*pax6A*), eye (*ovo*), pharynx (*foxA*) and also listed in Supplementary Table 3.

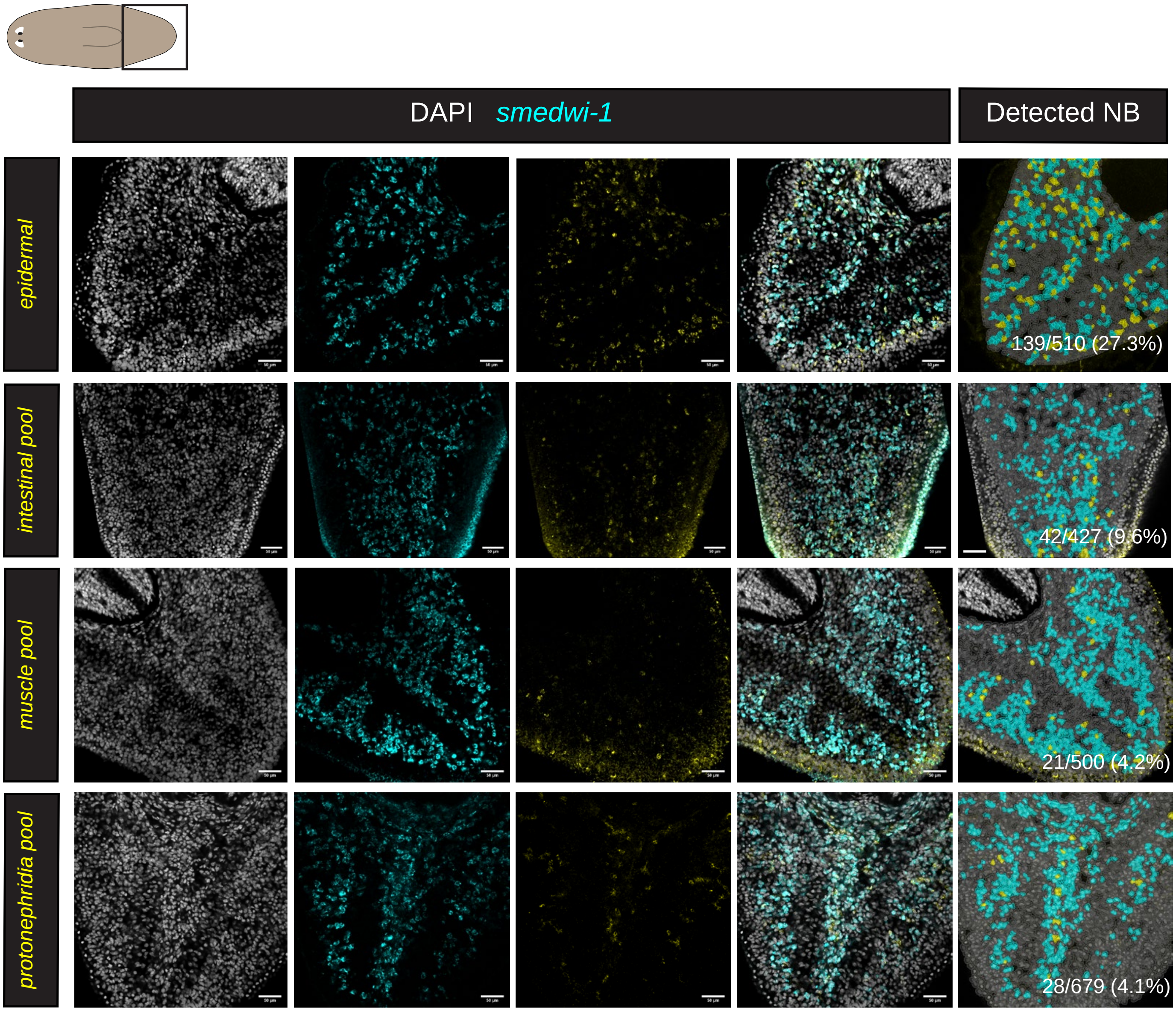
DAPI smedwi-1
Detected NB
epidermal
intestinal pool
muscle pool
protonephridia pool
139/510 (27.3%)
42/427 (9.6%)
21/500 (4.2%)
28/679 (4.1%)

Sup Fig. 16

Supplementary Figure 16. Specialized neoblasts of various classes are scattered throughout the planarian tail.

Whole-mount FISH images from the planarian tail showing specialized neoblasts (SpNBs) intermingled with non-self neoblasts. Epidermal and intestinal panels are the same as images shown in Figure 2B. Numbers represent the proportion of a SpNB class compared to all detected neoblasts. Scale bar 50 µm. Genes for tissue-specific FSTF pools: epidermis (*soxP-3*), intestine (*hnf-4*, *gata4/5/6-1*), protonephridia (*POU2/3*, *six-1/2-2*), muscle (*myoD*, *snail*) and also listed in Supplementary Table 3.

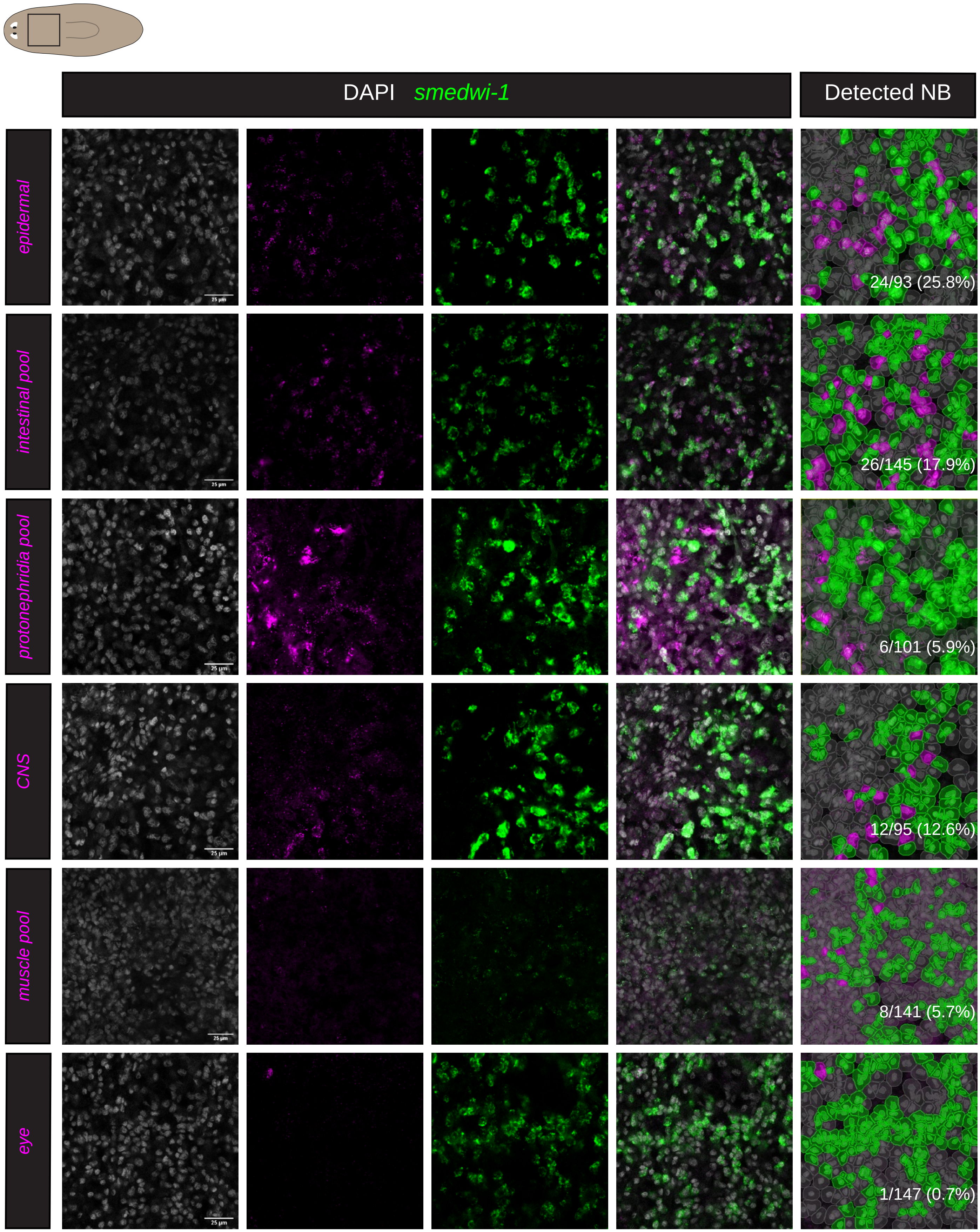
DAPI smedwi-1
Detected NB
epidermal
intestinal pool
protonephridia pool
CNS
muscle pool
eye
24/93 (25.8%)
26/145 (17.9%)
6/101 (5.9%)
12/95 (12.6%)
8/141 (5.7%)
1/147 (0.7%)

Sup Fig. 17

Supplementary Figure 17. The pre-pharyngeal region of the planarian contains a scattered-distribution of specialized neoblasts.
Whole-mount FISH images from the planarian pre-pharyngeal (above pharynx/below eyes) showing specialized neoblasts intermingled with non-self neoblasts. Genes for tissue-specific FSTF pools listed in Supplementary Table 3. Numbers represent the proportion of a specialized neoblast class compared to all detected neoblasts. Genes for tissue-specific FSTF pools: epidermis (*soxP-3*), intestine (*hnf-4*, *gata4/5/6-1*), protonephridia (*POU2/3*, *six-1/2-2*), muscle (*myoD, snail*), CNS (*pax6A*), eye (*ovo*) and also listed in Supplementary Table 3.

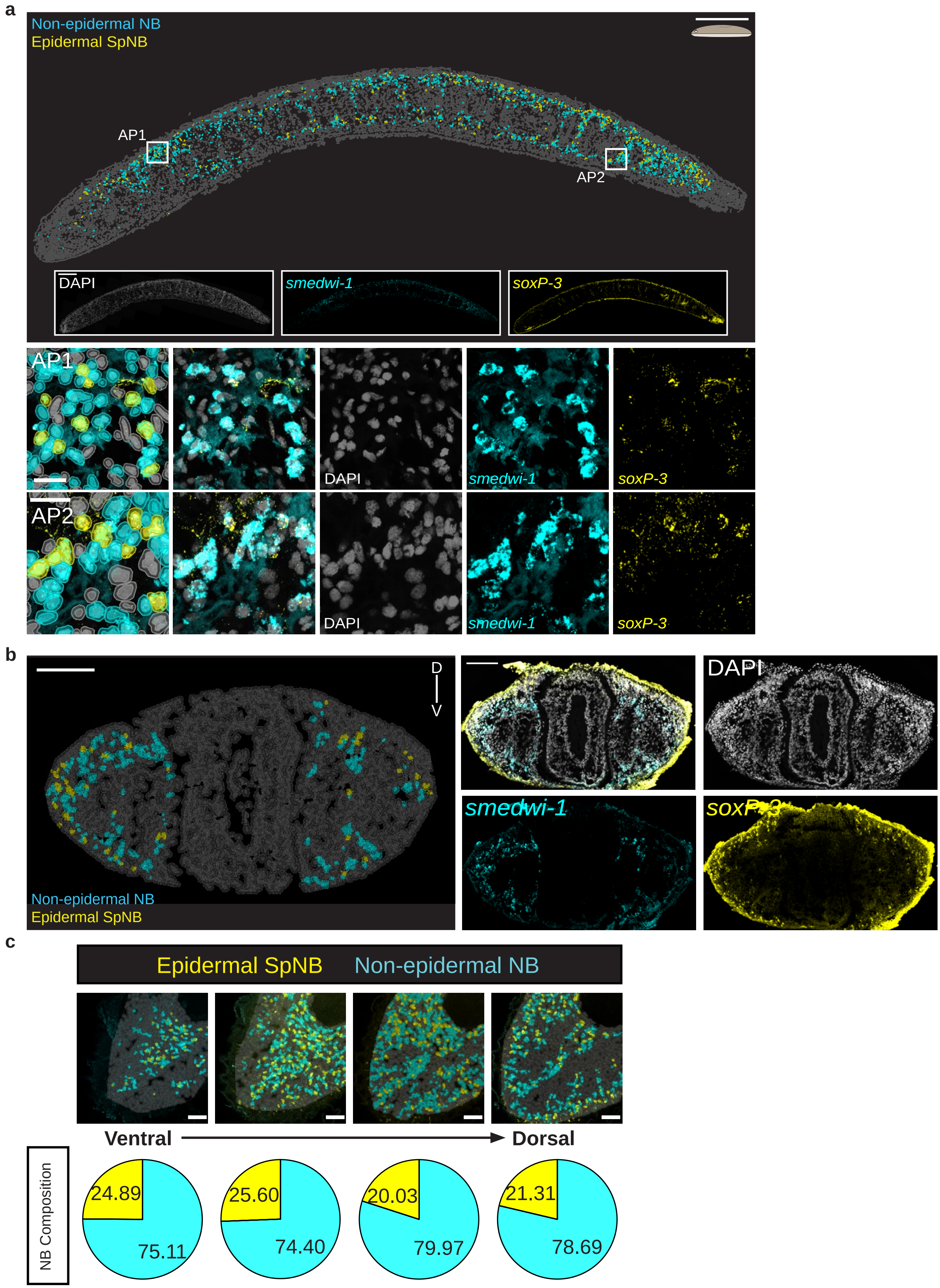
a
Non-epidermal NB
Epidermal SpNB
AP1
AP2
DAPI
smedwi-1
soxP-3
AP1
DAPI
smedwi-1
soxP-3
AP2
DAPI
smedwi-1
soxP-3
b
D
V
Non-epidermal NB
Epidermal SpNB
DAPI
smedwi-1
soxP-3
c
Epidermal SpNB
Non-epidermal NB
Ventral
Dorsal
NB Composition
24.89
75.11
25.60
74.40
20.03
79.97
21.31
78.69

Sup Fig. 18

Supplementary Figure 18. A scattered pattern of neoblast fate choice exists across multiple planarian body axes.
(a) Epidermal specialized neoblasts (SpNBs) are distributed amongst non-self neoblasts across the anteroposterior axis. Thin sagittal section. Scale bars, (top) 200 µm, (insets) 250 µm, (AP1 and AP2) 20 µm. (b) Epidermal SpNBs are distributed amongst non-self neoblast across the dorsoventral axis. Thin transverse section. Scale bars, 100 µm. (c) Cross sections from whole-mount FISH depicting distribution of epidermal SpNBs and non-self neoblasts in multiple z-sections from the ventral surface (left) moving dorsally (right). Pie graphs depict the percentage of identified neoblasts within each of the z-sections above. N = 225 (first), 461 (second), 749 (third), 474 (fourth). Scale bars, 50 µm. Source data are provided as a Source Data file.

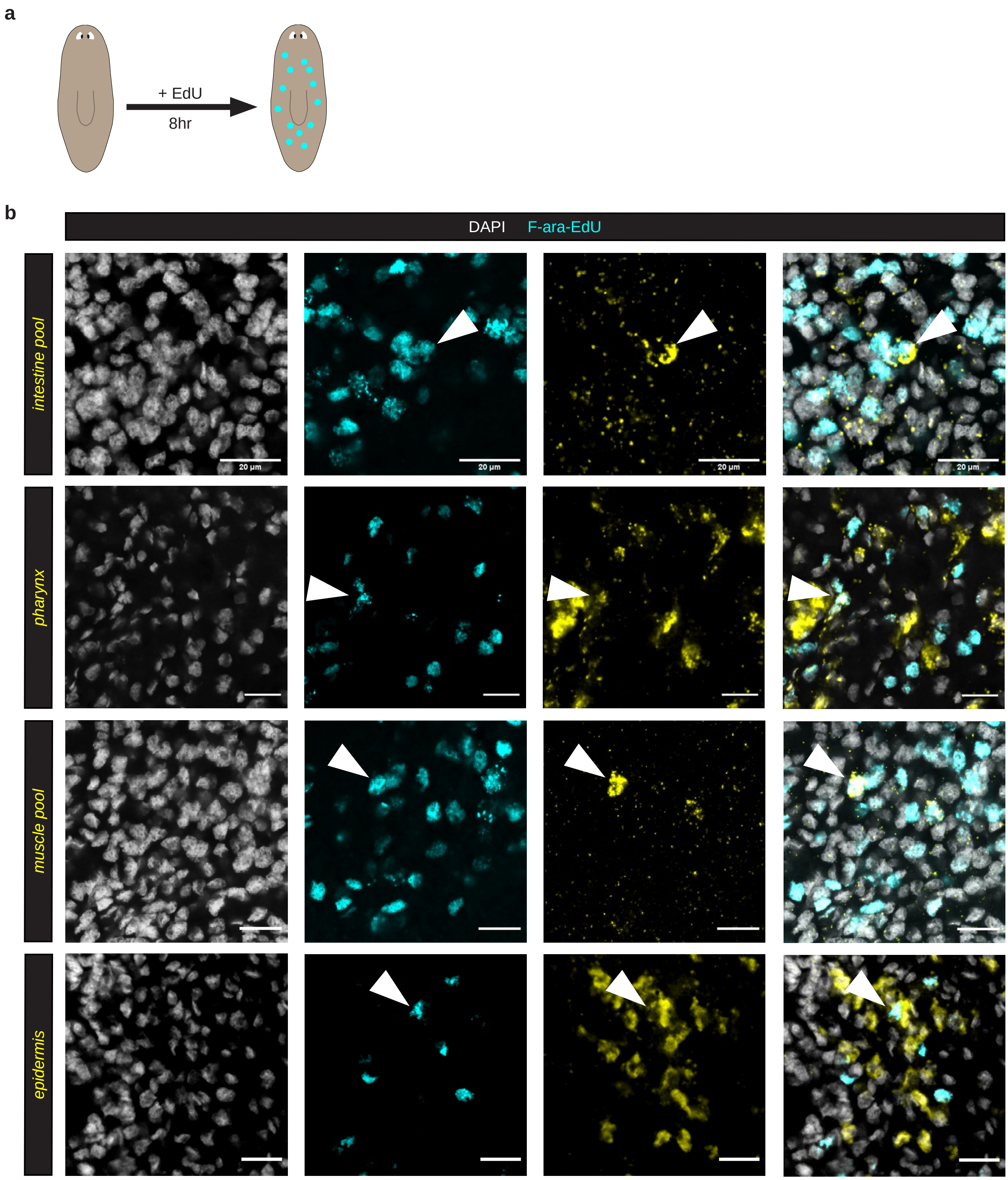

a
+ EdU
8hr
b
DAPI
F-ara-EdU
intestine pool
pharynx
muscle pool
epidermis
20 µm
20 µm
20 µm
20 µm

Sup Fig. 19

Supplementary Figure 19. Initial neoblast fate choices are not made in distinct local clusters in intact planarians.
(a) Cartoon showing how 8 hour EdU pulse in intact animals was used to mark neoblasts actively/recently replicating DNA. (b) Whole-mount FISH showing EdU^+ specialized neoblasts (white arrows) in clustered proximity to EdU^- neoblasts. Scale bar, (intestine pool) 20 µm, (pharynx, muscle pool, epidermis) 10 µm. Genes for tissue-specific FSTF pools: epidermis (*soxP-3*), intestine (*hnf-4*, *gata4/5/6-1*), muscle (*myoD*, *snail*), pharynx (*foxA*) and also listed in Supplementary Table 3.

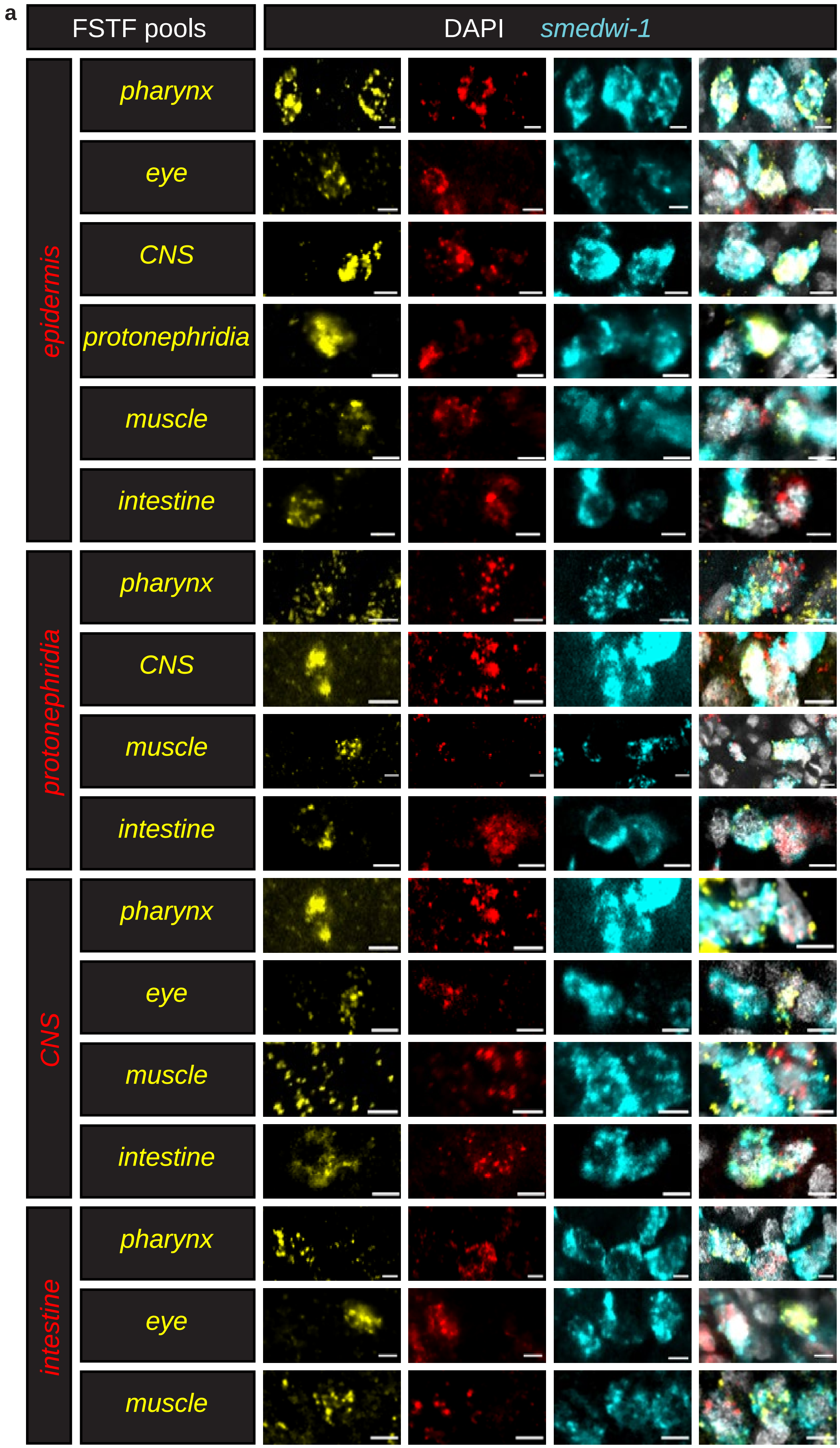
a
FSTF pools
DAPI
smedwi-1
epidermis
pharynx
eye
CNS
protonephridia
muscle
intestine
protonephridia
pharynx
CNS
muscle
intestine
CNS
pharynx
eye
muscle
intestine
intestine
pharynx
eye
muscle

Sup Fig. 20

b

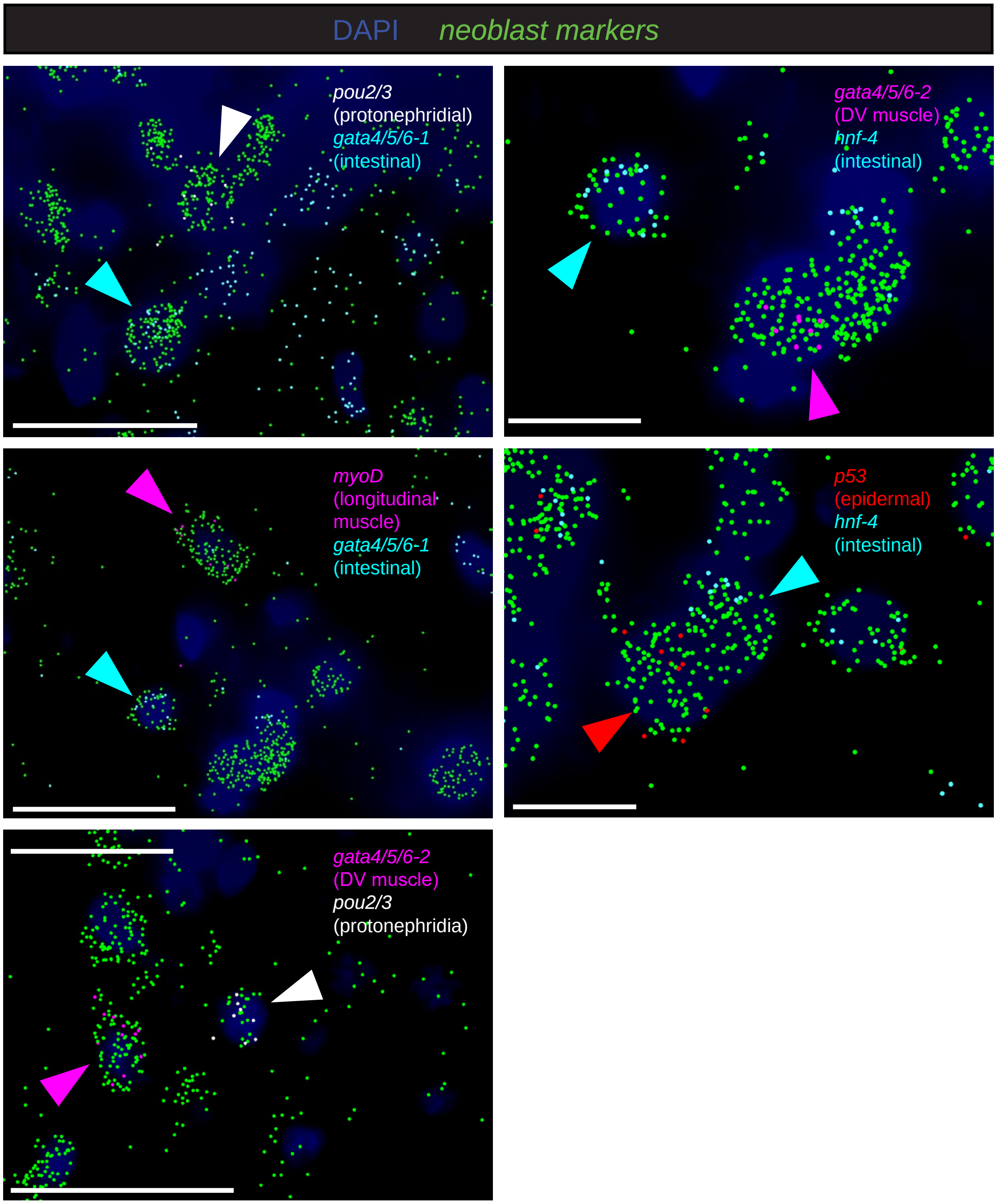

DAPI neoblast markers
pou2/3
(protonephridial)
gata4/5/6-1
(intestinal)
gata4/5/6-2
(DV muscle)
hnf-4
(intestinal)
myoD
(longitudinal
muscle)
gata4/5/6-1
(intestinal)
p53
(epidermal)
hnf-4
(intestinal)
gata4/5/6-2
(DV muscle)
pou2/3
(protonephridia)

Supplementary Figure 20. Specialized neoblasts of different fates are found in clustered proximity to one another in the intact animal.
(a) Whole-mount FISH images showing specialized neoblasts of various classes in close proximity to specialized neoblasts of a different fate. Images shown are the same as in Figure 3C. FSTF pools described in Supplementary Table 3. Scale bar, 5 µm. Genes for tissue-specific FSTF pools: epidermis (*soxP-3*), intestine (*hnf-4, gata4/5/6-1*), protonephridia (*POU2/3, six-1/2-2*), muscle (*myoD, snail*), CNS (*pax6A*), eye (*ovo*), pharynx (*foxA*) and also listed in Supplementary Table 3. (b) MERFISH images showing specialized neoblasts of various classes in close proximity using single markers. Arrows depict identified specialized neoblasts with colors corresponding to fate located at the top right of each image. Scale bars, 25 µm (left column), 10 µm (right column).

a

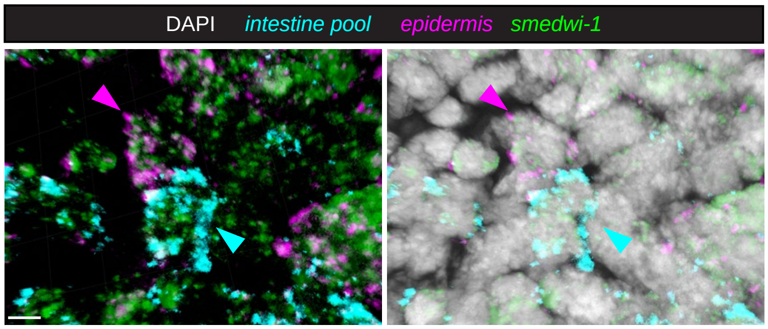

DAPI
intestine pool
epidermis
smedwi-1

b

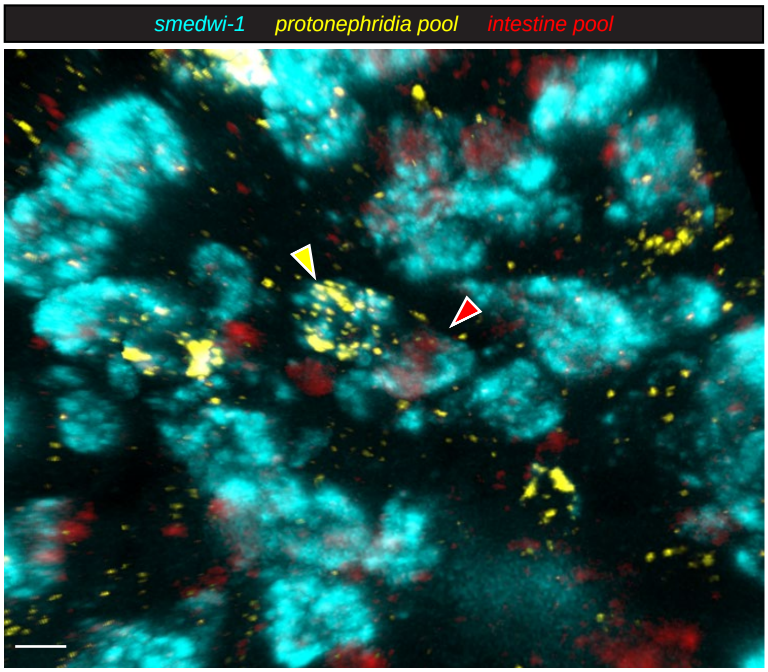

smedwi-1
protonephridia pool
intestine pool

Sup Fig. 21

Supplementary Figure 21. Three-dimensional images showing specialized neoblasts of different fates neighboring one another.
(a) Three-dimensional signal projection of whole-mount FISH showing intestinal and epidermal-specialized neoblasts neighboring one another. Arrowheads indicate specialized neoblasts. Scale bar, 5 µm. (b) Three-dimensional snapshot from Movie S8 showing protonephridia and intestinal/specialized neoblasts in neighboring proximity. Arrowheads indicate specialized neoblasts. Scale bar, 5 µm. Genes for tissue-specific FSTF pools: epidermis (*soxP-3*), intestine (*hnf-4, gata4/5/6-1*), protonephridia (*POU2/3, six-1/2-2*) and also listed in Supplementary Table 3.

a

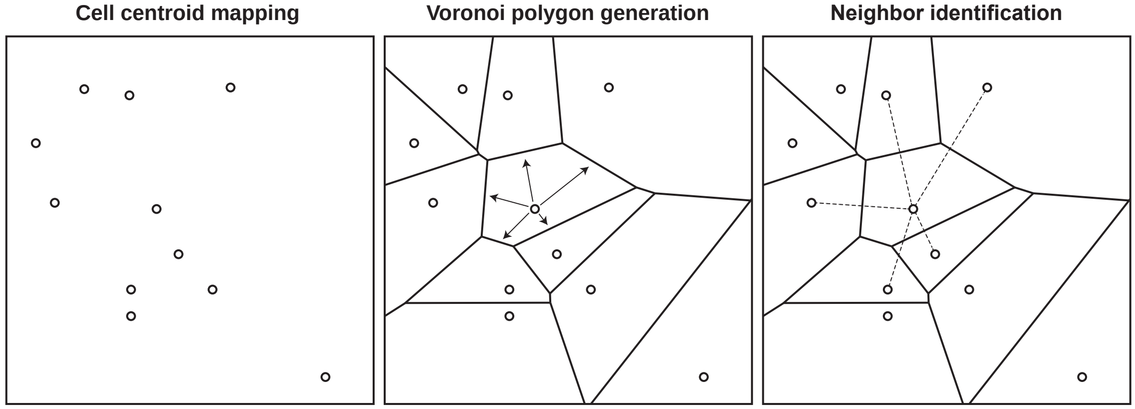

Cell centroid mapping
Voronoi polygon generation
Neighbor identification

b

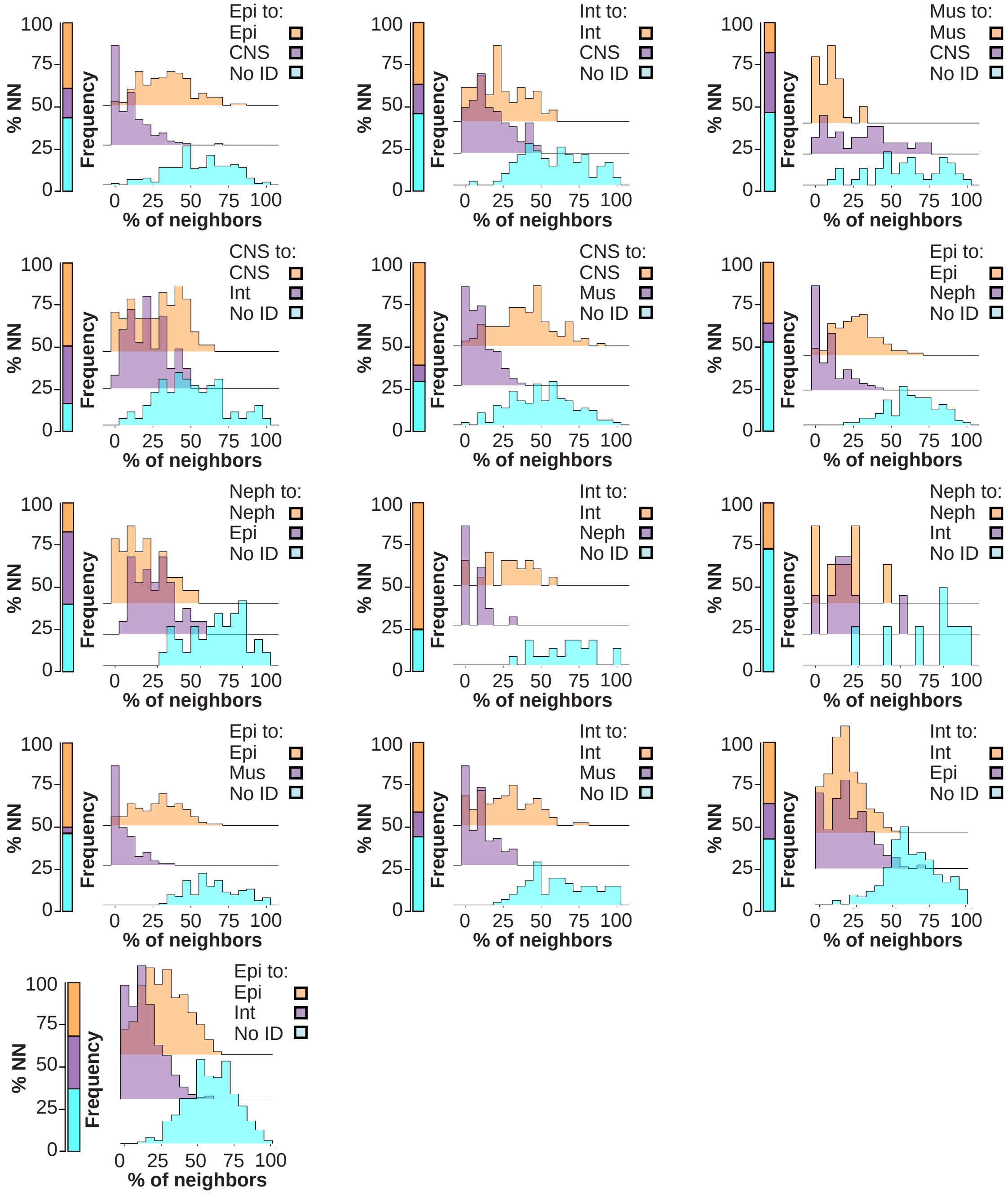

% NN
Frequency
% of neighbors
100
75
50
25
0
Epi to:
Epi
CNS
No ID
Int to:
Int
CNS
No ID
Mus to:
Mus
CNS
No ID
CNS to:
CNS
Int
No ID
CNS to:
CNS
Mus
No ID
Epi to:
Epi
Neph
No ID
Neph to:
Neph
Epi
No ID
Int to:
Int
Neph
No ID
Neph to:
Neph
Int
No ID
Epi to:
Epi
Mus
No ID
Int to:
Int
Mus
No ID
Int to:
Int
Epi
No ID
Epi to:
Epi
Int
No ID

Sup Fig. 22

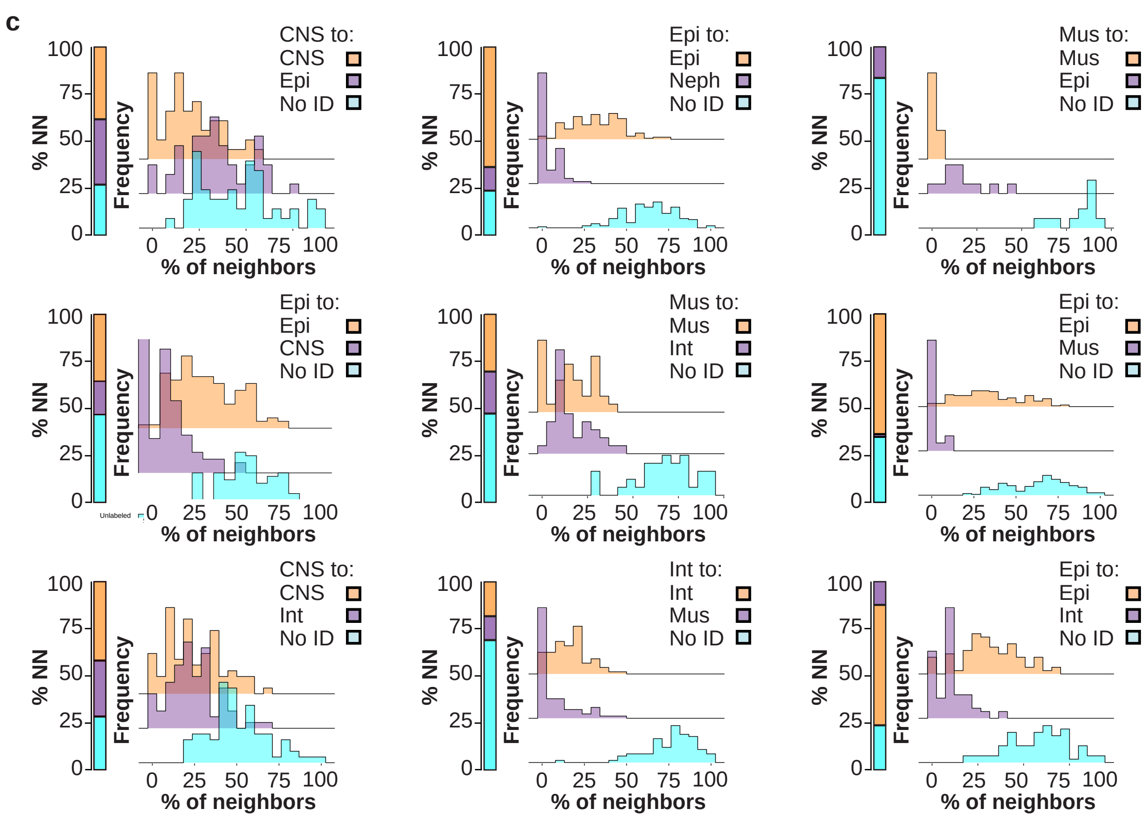
c
CNS to:
CNS
Epi
No ID
Epi to:
Epi
Neph
No ID
Mus to:
Mus
Epi
No ID
Epi to:
Epi
CNS
No ID
Mus to:
Mus
Int
No ID
Epi to:
Epi
Mus
No ID
CNS to:
CNS
Int
No ID
Int to:
Int
Mus
No ID
Epi to:
Epi
Int
No ID
% NN
Frequency
% of neighbors
Unlabeled

Sup Fig. 22

Supplementary Figure 22. Neighborhood composition analysis of specialized neoblasts.
(a) 2-dimensional conceptual model of neighborhood identification using Voronoi tessellation. Centroid positions of neoblasts are tessellated, and neoblast associated tessellations that share a border (or area in 3-dimensions) are identified as first-degree neighbors. The set of first-degree neighbors for a given neoblast position comprises its neighborhood. (B and C) Stacked bar plots represent percentage of nearest neighbors for sampled cells of a given specialized neoblast query class that are of labeled identities. Ridgeline plots represent percentage of cells in the Voronoi tessellation neighborhoods of sampled cells of a given specialized neoblast query class that are of labeled identities. Data generated from neoblasts within (b) pre-pharyngeal or (c) tail regions. N values provided in Supplementary Table 4. NN, nearest neighbor. SpNBs for: Mus, muscle; Epi, epidermal; CNS, central nervous system; Int, intestine; Neph, protonephridia. Source data are provided as a Source Data file.

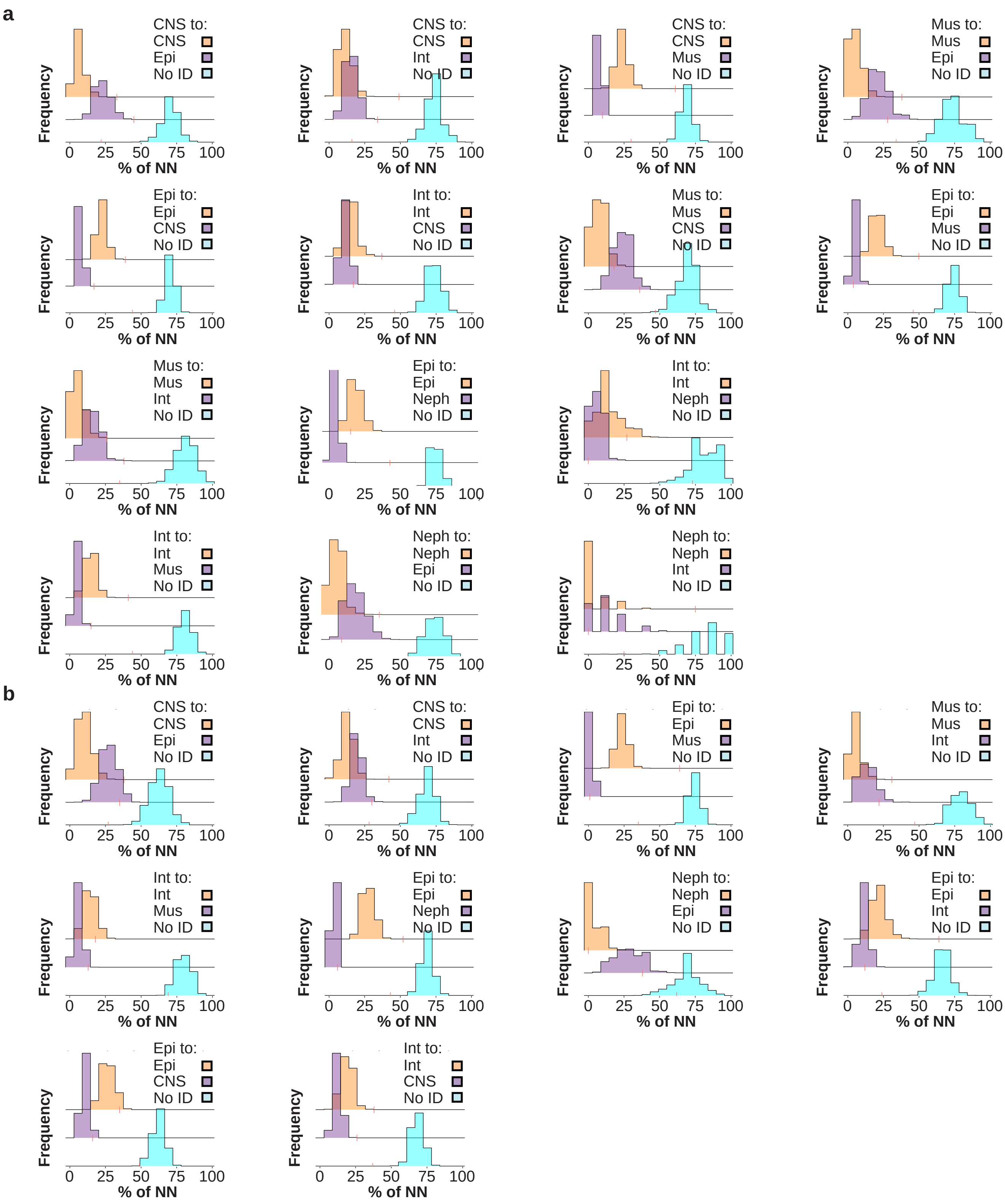
a
CNS to:
CNS
Epi
No ID
CNS to:
CNS
Int
No ID
CNS to:
CNS
Mus
No ID
Mus to:
Mus
Epi
No ID
Epi to:
Epi
CNS
No ID
Int to:
Int
CNS
No ID
Mus to:
Mus
CNS
No ID
Epi to:
Epi
Mus
No ID
Mus to:
Mus
Int
No ID
Epi to:
Epi
Neph
No ID
Int to:
Int
Neph
No ID
Int to:
Int
Mus
No ID
Neph to:
Neph
Epi
No ID
Neph to:
Neph
Int
No ID
b
CNS to:
CNS
Epi
No ID
CNS to:
CNS
Int
No ID
Epi to:
Epi
Mus
No ID
Mus to:
Mus
Int
No ID
Int to:
Int
Mus
No ID
Epi to:
Epi
Neph
No ID
Neph to:
Neph
Epi
No ID
Epi to:
Epi
Int
No ID
Epi to:
Epi
CNS
No ID
Int to:
Int
CNS
No ID
Frequency
0 25 50 75 100
% of NN

Sup Fig. 23

Supplementary Figure 23. Comparative analysis of randomized nearest neighbor identity composition to observed data

(A and B) Randomized nearest neighbor identity composition for a given query class (n=1,000 simulations) compared to observed nearest neighbor composition (red bar) from Supplementary Fig 22b,c for neoblasts from (a) pre-pharyngeal or (b) tail regions. NN, nearest neighbor. Specialized neoblasts for: Mus, muscle; Epi, epidermal; CNS, central nervous system; Int, intestine; Neph, protonephridia. Source data are provided as a Source Data file.

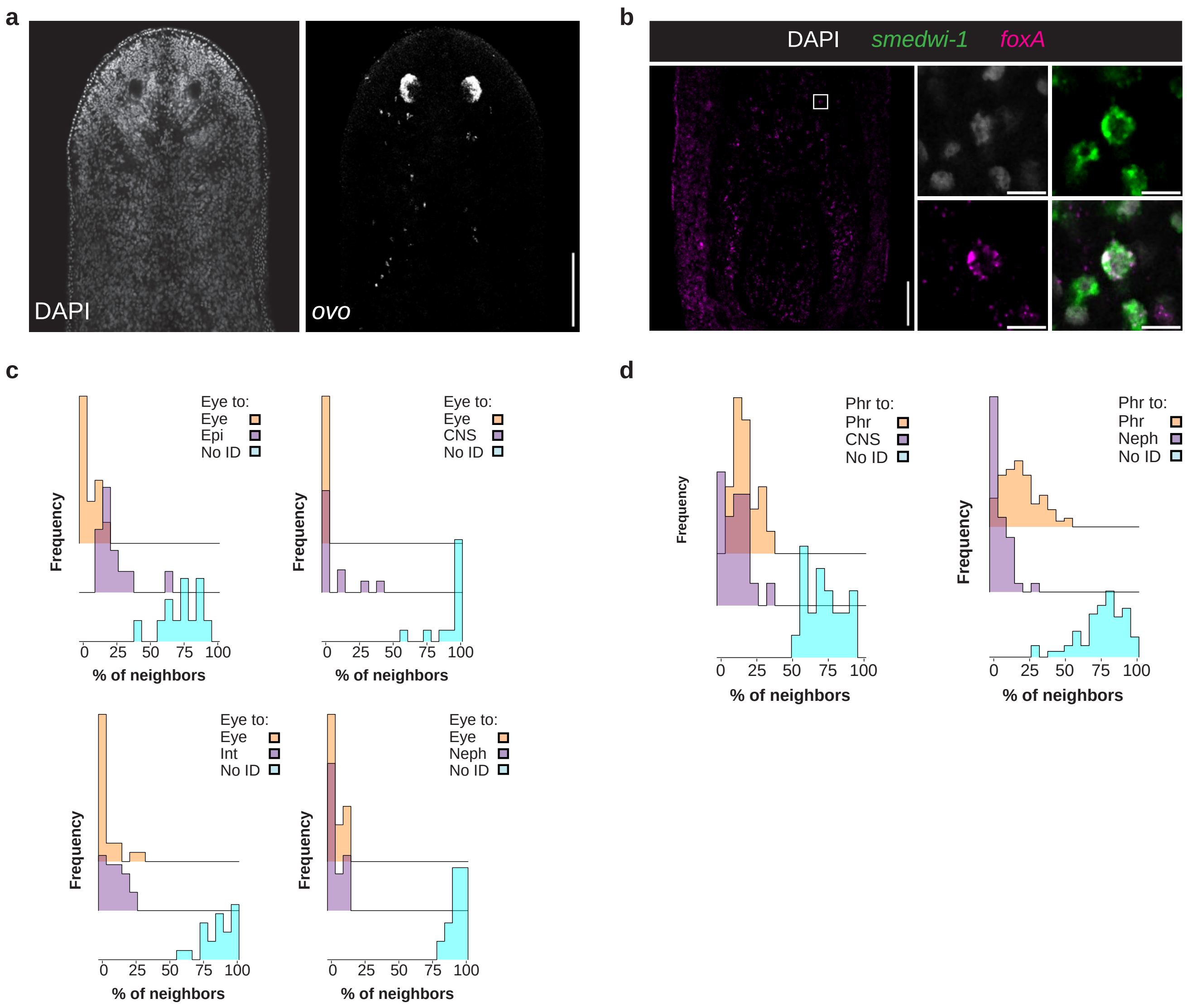
a
DAPI
ovo
b
DAPI smedwi-1 foxA
c
Eye to:
Eye
Epi
No ID
Frequency
0 25 50 75 100
% of neighbors
Eye to:
Eye
CNS
No ID
Frequency
0 25 50 75 100
% of neighbors
Eye to:
Eye
Int
No ID
Frequency
0 25 50 75 100
% of neighbors
Eye to:
Eye
Neph
No ID
Frequency
0 25 50 75 100
% of neighbors
d
Phr to:
Phr
CNS
No ID
Frequency
0 25 50 75 100
% of neighbors
Phr to:
Phr
Neph
No ID
Frequency
0 25 50 75 100
% of neighbors

Sup Fig. 24

Supplementary Figure 24. Eye and pharynx specialized neoblasts are intermingled with specialized neoblasts of different fate in homeostasis.
(a) FISH images of *ovo*+ cells in uninjured animals. Scale bar, 100 µm. (b) FISH images of *foxA+* pharyngeal specialized neoblasts in uninjured animals. (c) Ridgeline plots of Voronoi neighborhood identity composition for eye specialized neoblasts in Fig. 3j. (d) Ridgeline plots of Voronoi neighborhood identity composition for pharyngeal specialized neoblasts. N values for (c) and (d) available in Supplementary Table 4. Epi, epidermal; Int, intestinal; Neph, protonephridial; Phr, pharyngeal; CNS, neural. Source data are provided as a Source Data file.

a

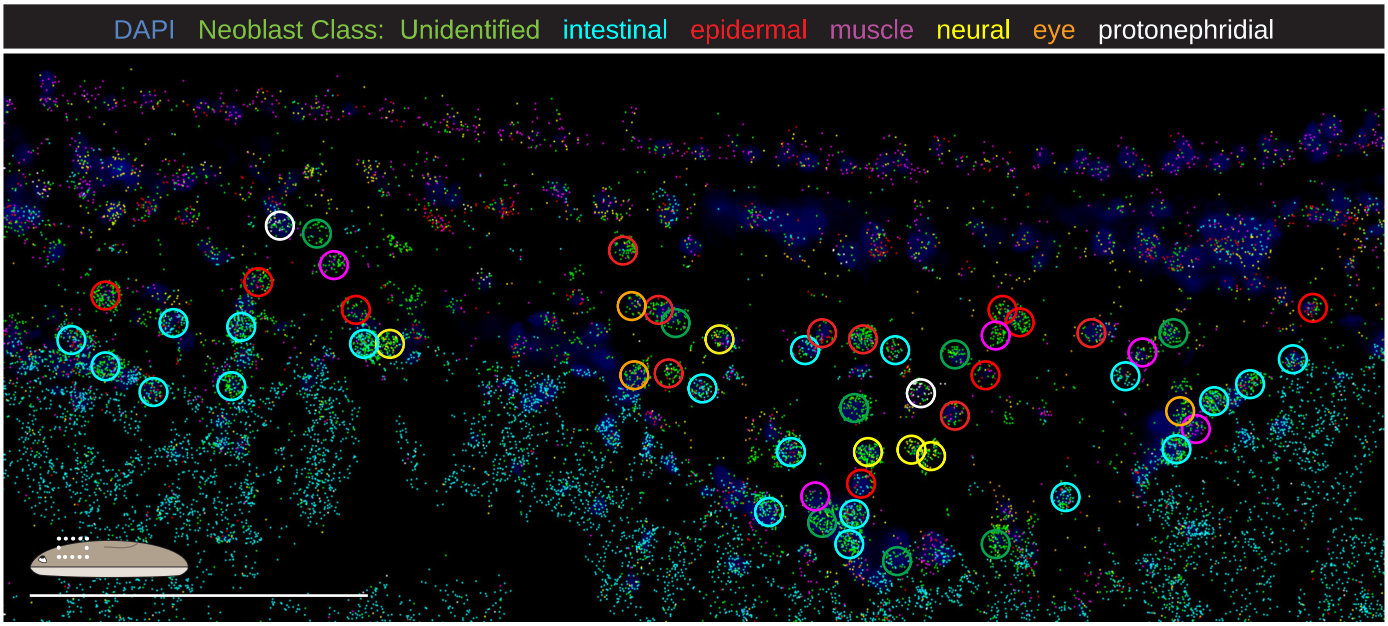

DAPI Neoblast Class: Unidentified intestinal epidermal muscle neural eye protonephridial

b

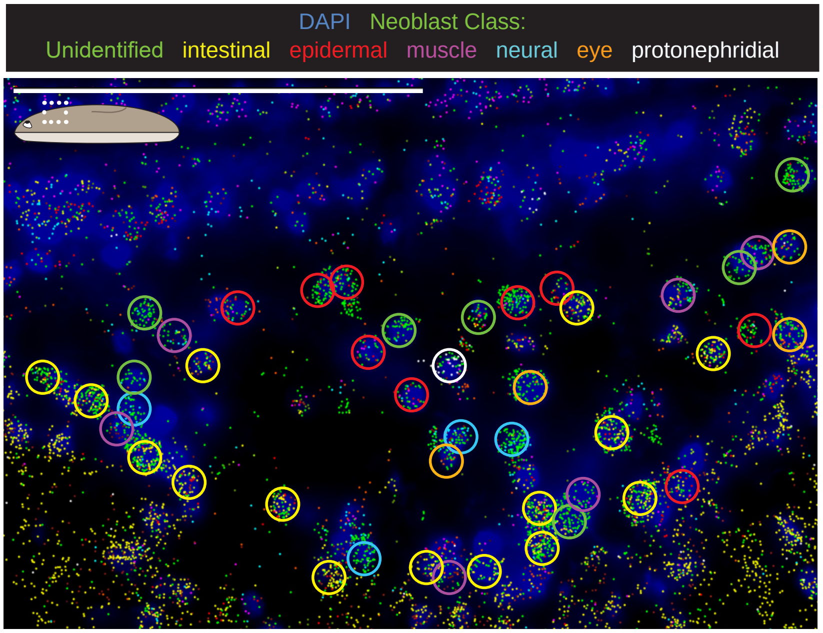

DAPI Neoblast Class:
Unidentified intestinal epidermal muscle neural eye protonephridial

Sup Fig. 25

Supplementary Figure 25. Fate specification is spatially intermingled in homeostasis
(A and B) Detection of multiple specialized neoblast (SpNB) classes by MERFISH in dorsal pre-pharyngeal regions of planarians. Colored circles represent identified SpNBs. (a) and (b) represent regions taken from independent sections. Scale bars, 100 µm.

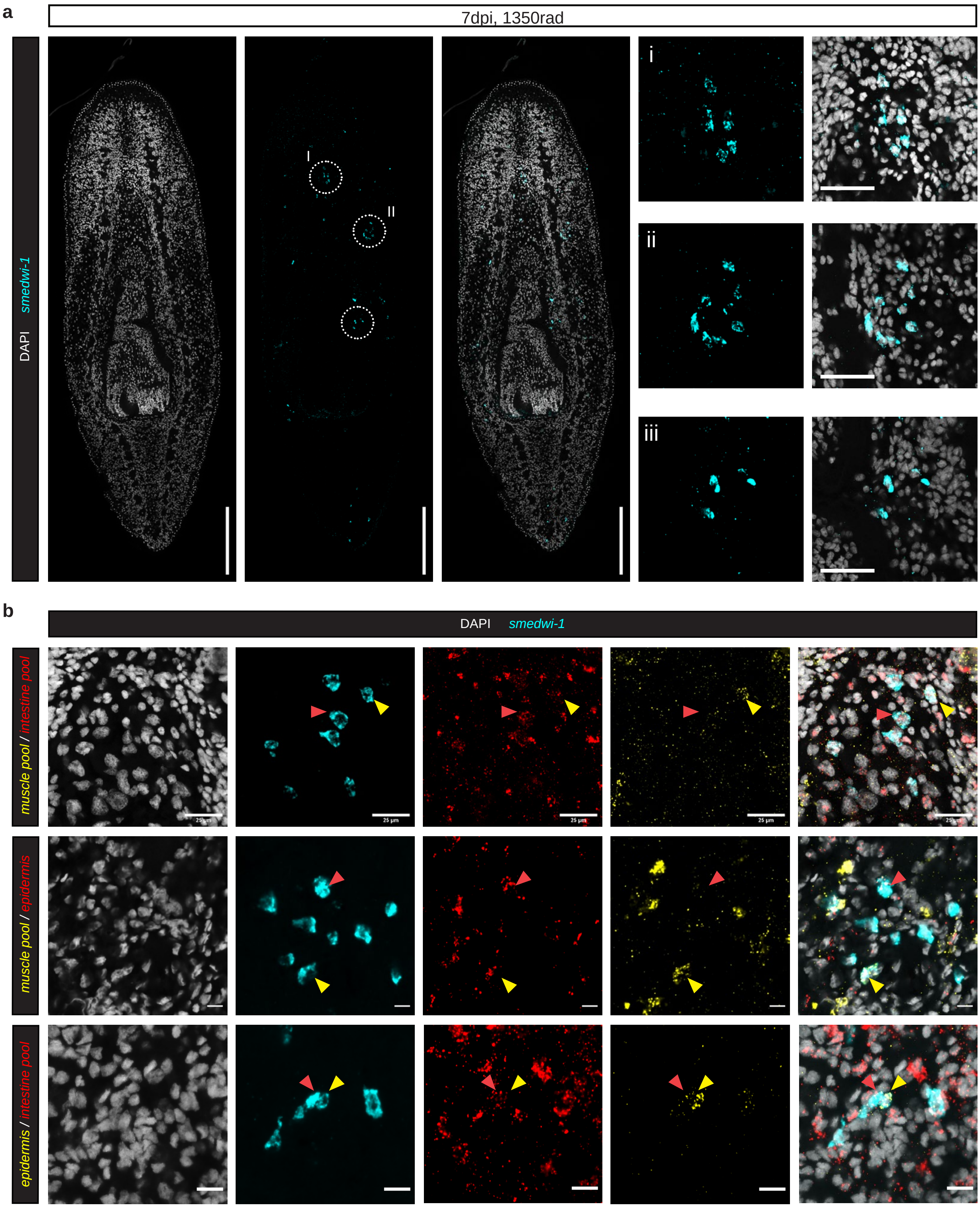
a
7dpi, 1350rad
DAPI smedwi-1
i
ii
iii
b
DAPI smedwi-1
muscle pool / intestine pool
muscle pool / epidermis
epidermis / intestine pool
25 µm

Sup Fig. 26

Supplementary Figure 26. Fate specification in neoblast colonies is spatially intermingled. (a) Subtotal irradiation in animals depicting neoblast colonies. Scale bar, 200µm. Image for (i) are the same as in Figure 4b. (b) Neoblast colonies from irradiated animals (subtotal) with identified specialized neoblasts of diverse fates within the same colony. Arrowheads indicate identified specialized neoblasts. Identified neoblast colonies were located ventrally in animals. Scale bars, (top) 25 µm, (middle) 10 µm, (bottom) 15 µm. Images are the same as in Figure 4c. Genes for tissue-specific FSTF pools: epidermis (*soxP-3*), intestine (*hnf-4, gata4/5/6-1*), muscle (*myoD, snail*) and also listed in Supplementary Table 3.

a

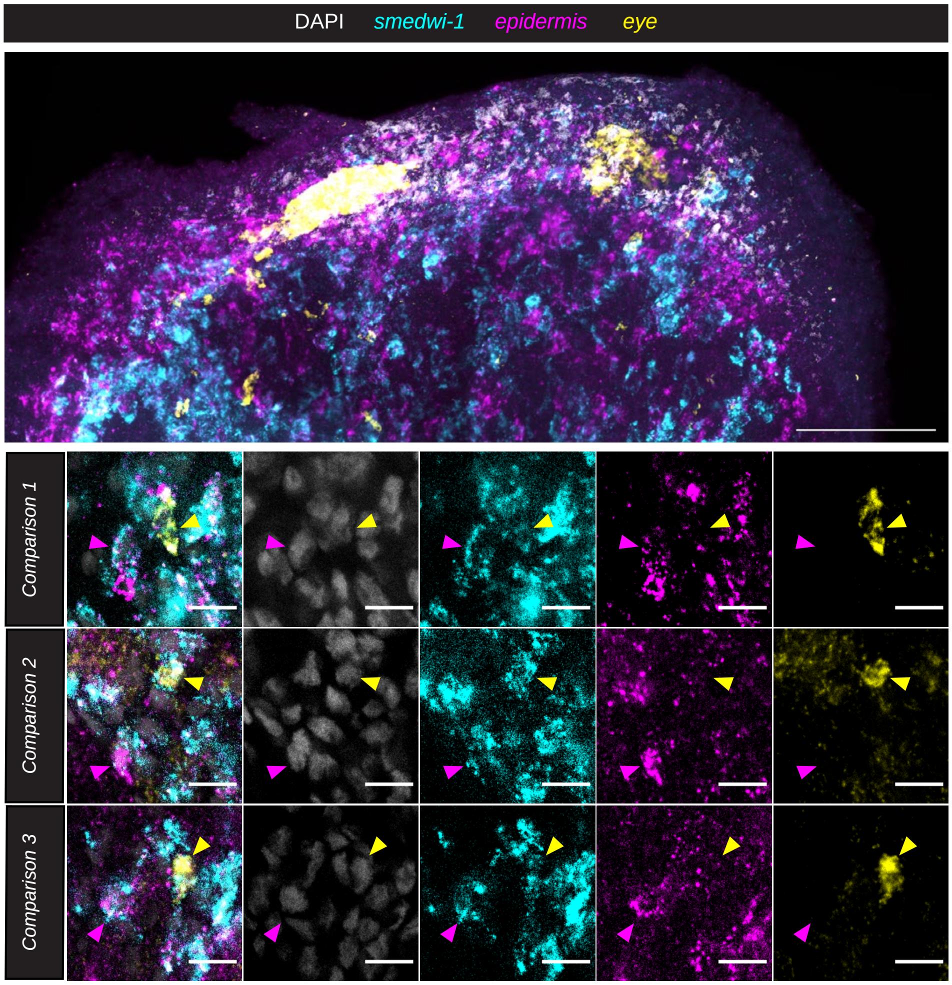

DAPI *smedwi-1* *epidermis* *eye*
Comparison 1
Comparison 2
Comparison 3

b

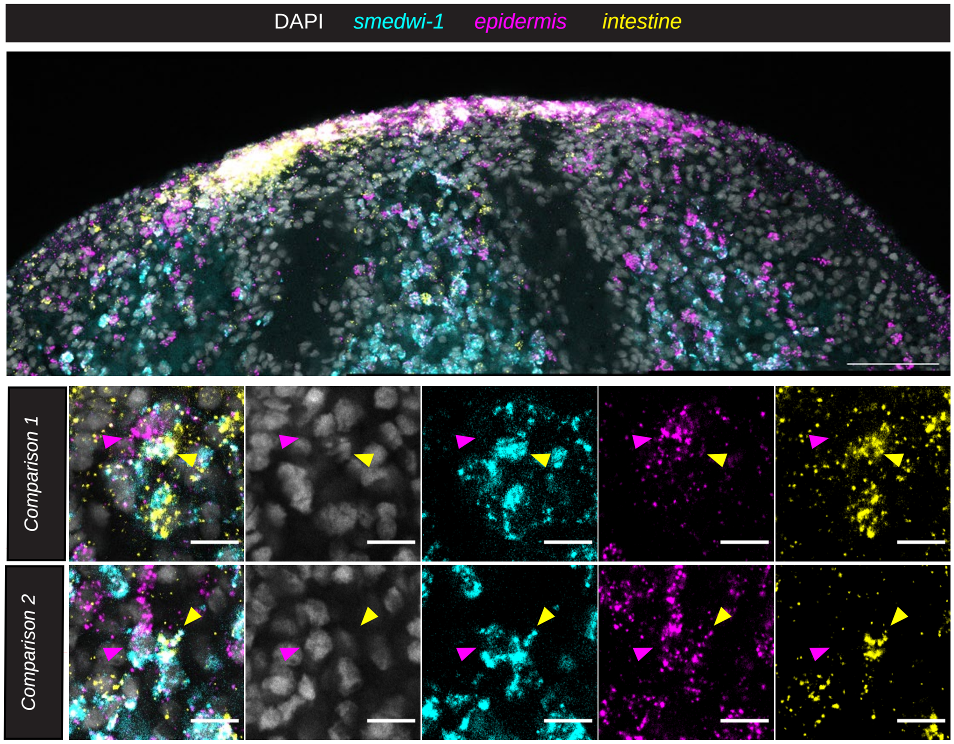

DAPI *smedwi-1* *epidermis* *intestine*
Comparison 1
Comparison 2

Sup Fig. 27

c

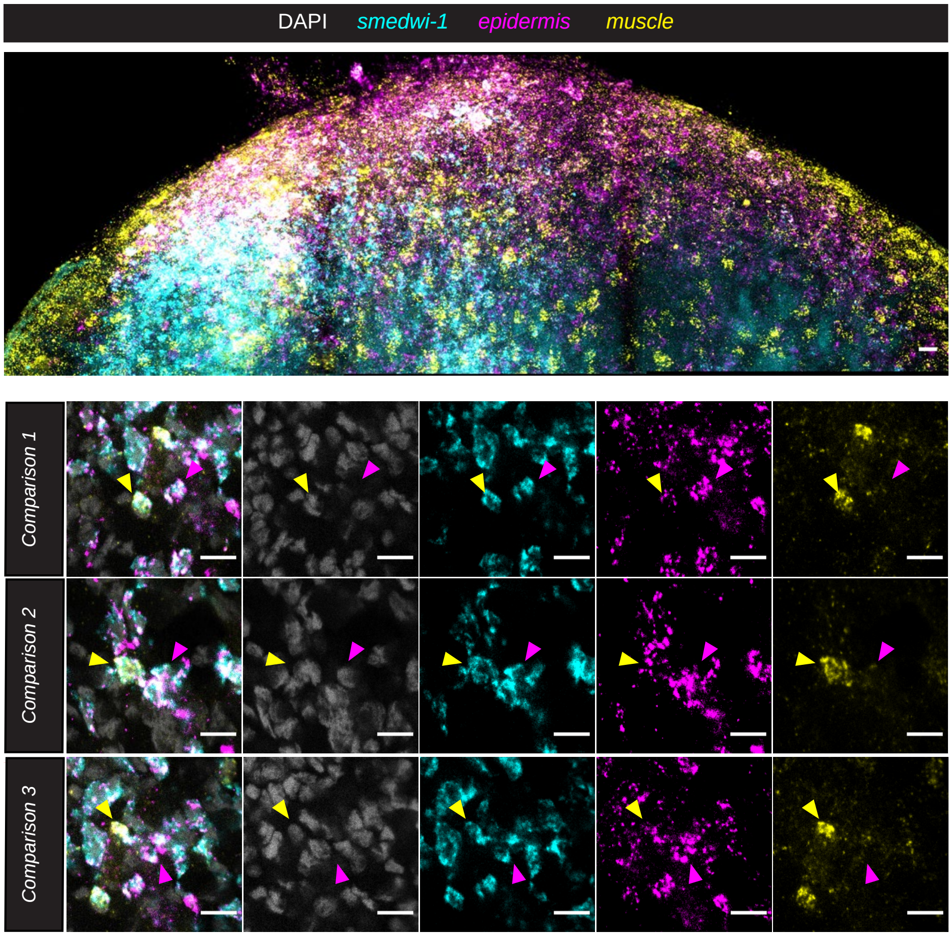
DAPI smedwi-1 epidermis muscle
Comparison 1
Comparison 2
Comparison 3

d

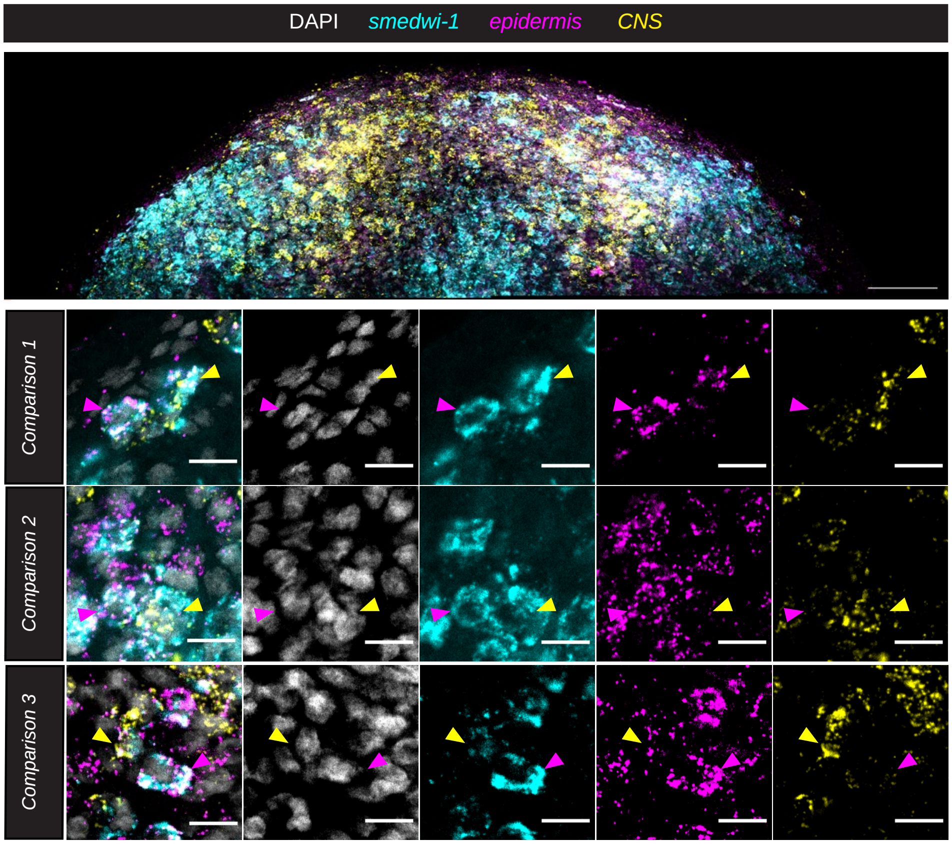
DAPI smedwi-1 epidermis CNS
Comparison 1
Comparison 2
Comparison 3

Sup Fig. 27

e

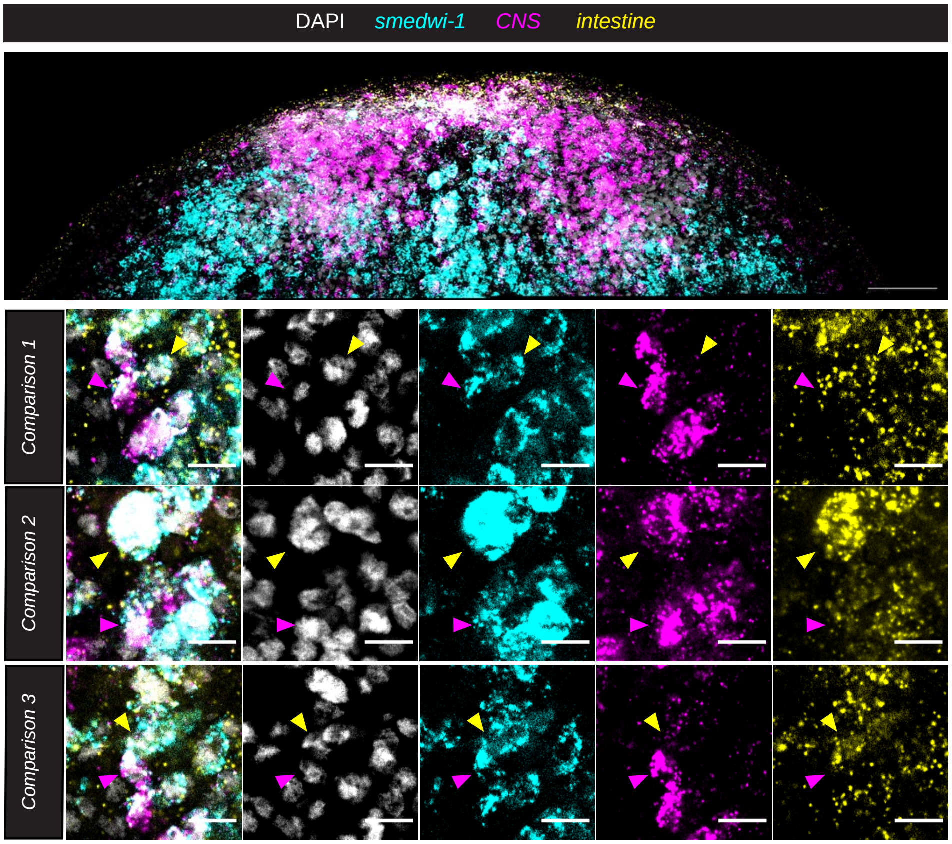

DAPI smedwi-1 CNS intestine
Comparison 1
Comparison 2
Comparison 3

f

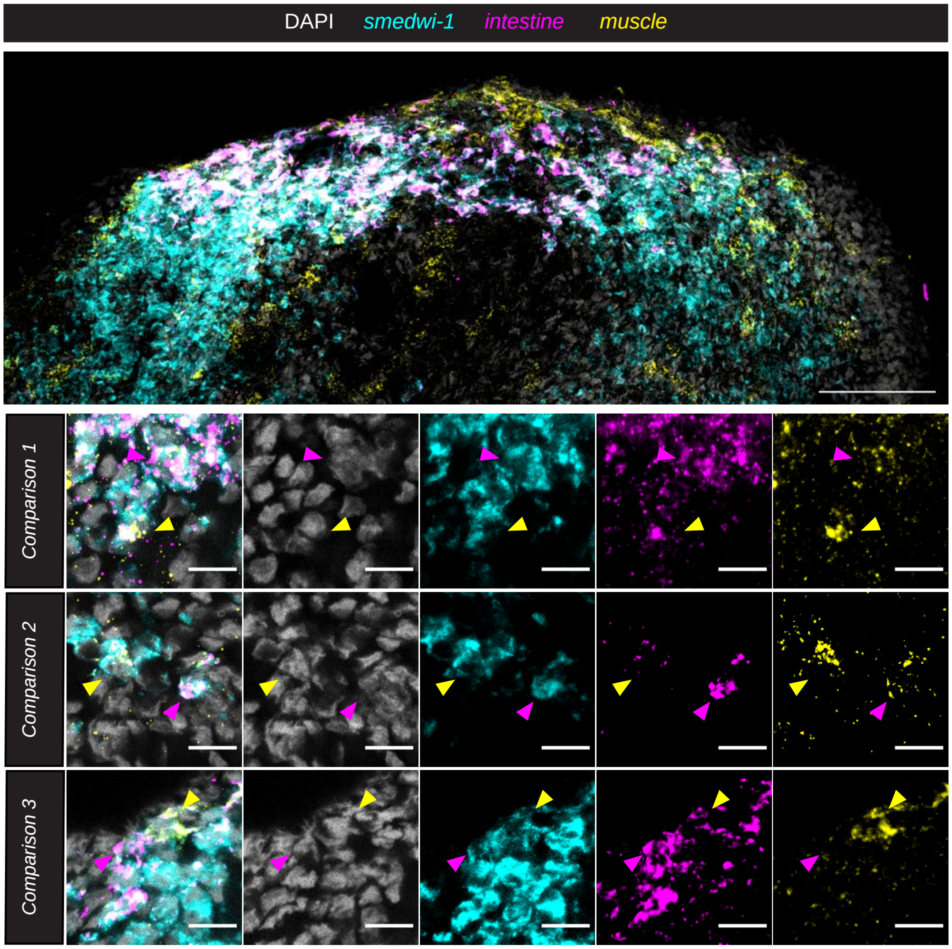

DAPI smedwi-1 intestine muscle
Comparison 1
Comparison 2
Comparison 3

Sup Fig. 27

Supplementary Figure 27. Fate specification is spatially intermingled in regeneration
(A-F) Specialized neoblasts of different fates are located in close proximity in anterior-facing wounds 72 hours post amputation. Pairwise comparisons for (a) eye and epidermal SpNBs, (b) epidermal and intestinal SpNBs, (c) epidermal and muscle SpNBs, (d) epidermal and CNS SpNBs, (e) CNS and intestinal SpNBs, and (f) intestinal and muscle SpNBs. Top images depict wound sites. Arrows depict identified SpNBs. Bottom images represent additional examples of identified SpNB pairs. Scale bars, top images 50 µm, bottom images 20 µm. Genes for tissue-specific FSTF pools: epidermis (*soxP-3*), intestine (*hnf-4*, *gata4/5/6-1*), muscle (*myoD*, *snail*), CNS (*pax6A*), eye (*ovo*) and also listed in Supplementary Table 3.

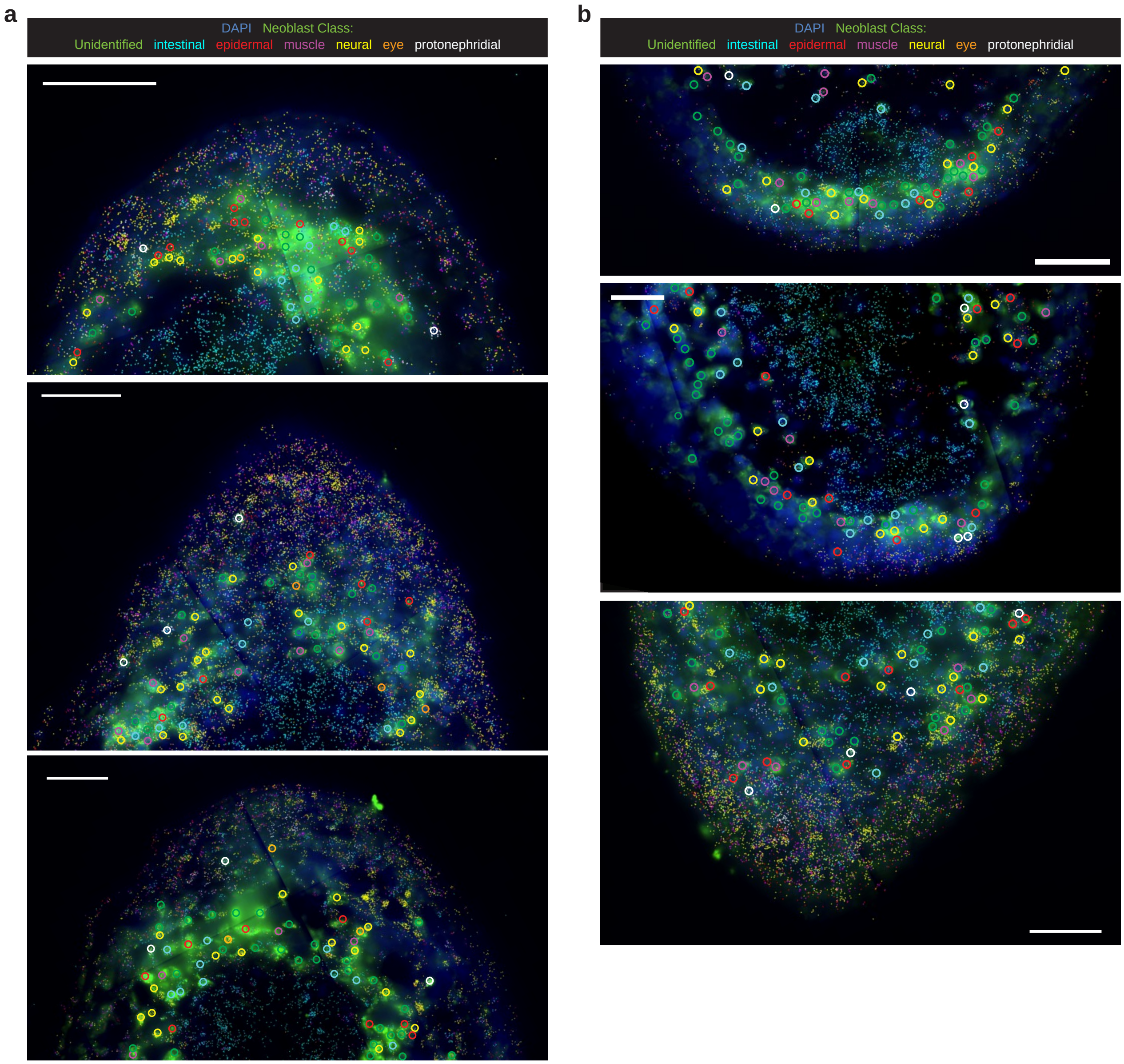
a
DAPI Neoblast Class:
Unidentified intestinal epidermal muscle neural eye protonephridial
b
DAPI Neoblast Class:
Unidentified intestinal epidermal muscle neural eye protonephridial

Sup Fig. 28

Supplementary Figure 28. Fate specification is spatially intermingled in regeneration by MERFISH.
(a-b) Detection of multiple specialized neoblast classes by MERFISH in anterior-facing wounds (a) and posterior-facing wounds (b). Each image represents an independent animal. Scale bars, 100 µm.

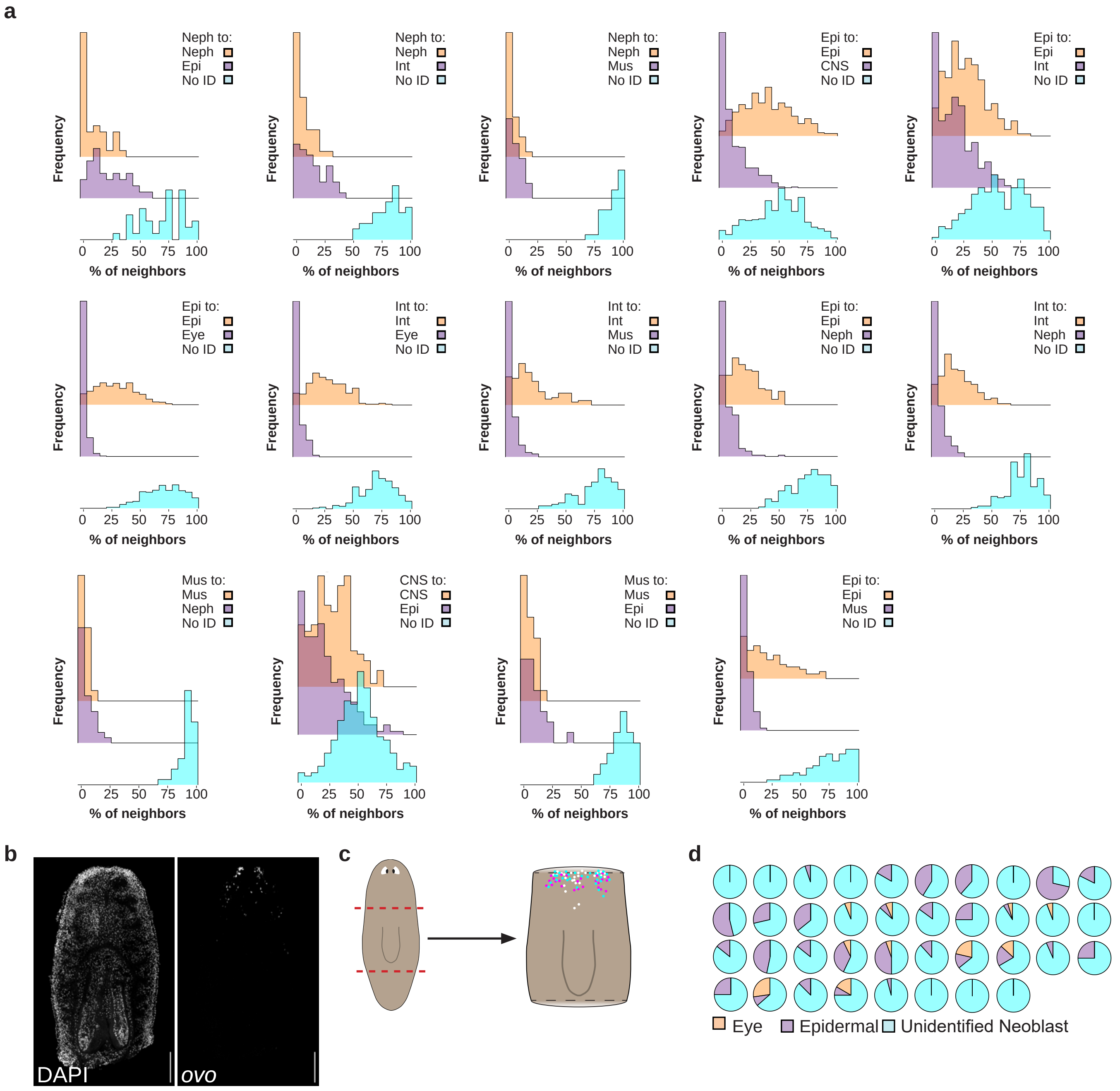
a
Neph to: Neph Epi No ID
Neph to: Neph Int No ID
Neph to: Neph Mus No ID
Epi to: Epi CNS No ID
Epi to: Epi Int No ID
Epi to: Epi Eye No ID
Int to: Int Eye No ID
Int to: Int Mus No ID
Epi to: Epi Neph No ID
Int to: Int Neph No ID
Mus to: Mus Neph No ID
CNS to: CNS Epi No ID
Mus to: Mus Epi No ID
Epi to: Epi Mus No ID
Frequency
0 25 50 75 100
% of neighbors
b
DAPI
ovo
c
d
Eye
Epidermal
Unidentified Neoblast

Sup Fig. 29

Supplementary Figure 29. Specialized neoblasts of different fates are spatially intermingled in regeneration.

(a) Ridgeline plots represent percentage of cells in the Voronoi tessellation neighborhoods of sampled cells of a given specialized neoblast query class that are of labeled identities. N values available in Supplementary Table 4. (b) FISH images of *ovo*+ cells in trunk fragments after the amputation described above. Scale bar, 100 µm. (c) Cartoon depicting the location of eye specialized neoblasts in trunk fragments after amputation of the head and tail. Colored circles represent eye specialized neoblast mapping from 3 independent animals. (d) Voronoi neighborhood identity composition of 38 eye specialized neoblasts. Data taken from Epi and Eye comparison in (a). Neph, protonephridial; Epi, epidermal; Int, intestinal; CNS, neural; Mus, muscle. Source data are provided as a Source Data file.

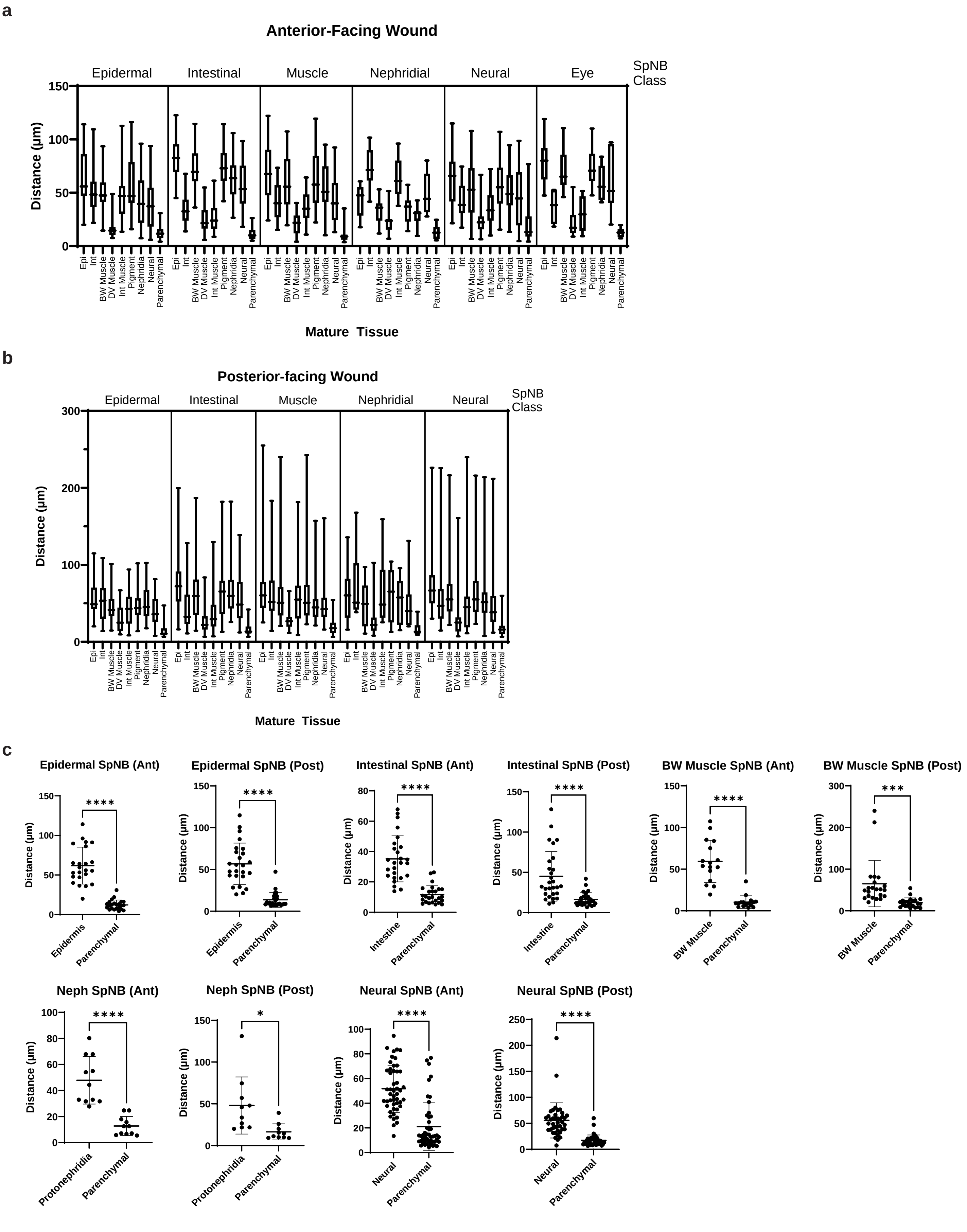
a
Anterior-Facing Wound
Epidermal
Intestinal
Muscle
Nephridial
Neural
Eye
SpNB Class
Distance (µm)
0
50
100
150
Epi
Int
BW Muscle
DV Muscle
Int Muscle
Pigment
Nephridia
Neural
Parenchymal
Mature Tissue
b
Posterior-facing Wound
Epidermal
Intestinal
Muscle
Nephridial
Neural
SpNB Class
Distance (µm)
0
100
200
300
Mature Tissue
c
Epidermal SpNB (Ant)
Epidermal SpNB (Post)
Intestinal SpNB (Ant)
Intestinal SpNB (Post)
BW Muscle SpNB (Ant)
BW Muscle SpNB (Post)
Neph SpNB (Ant)
Neph SpNB (Post)
Neural SpNB (Ant)
Neural SpNB (Post)
Distance (µm)
****
***
*
Epidermis
Intestine
BW Muscle
Protonephridia
Neural
Parenchymal

Sup Fig. 30

d

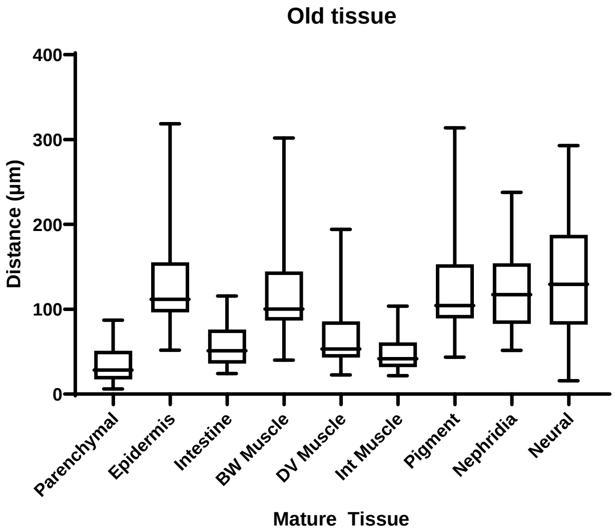

Old tissue
Distance (µm)
400
300
200
100
0
Parenchymal
Epidermis
Intestine
BW Muscle
DV Muscle
Int Muscle
Pigment
Nephridia
Neural
Mature Tissue

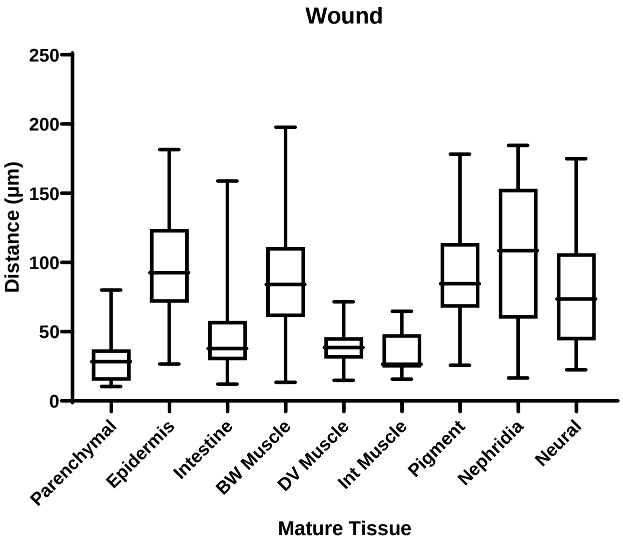

Wound
Distance (µm)
250
200
150
100
50
0
Parenchymal
Epidermis
Intestine
BW Muscle
DV Muscle
Int Muscle
Pigment
Nephridia
Neural
Mature Tissue

Sup Fig. 30

Supplementary Figure 30. Distance measurements of SpNBs to mature tissues in regeneration.

(a) Distance measurements of specified SpNBs to mature tissues in anterior- (a) or posterior- (b) facing wounds. N = 23 (Epidermal), 26 (Intestinal), 16 (Muscle), 11 (Protonephridial), 48 (Neural), 7 (Eye) in (a). N = 28 (Epidermal), 28 (Intestinal), 22 (Muscle), 10 (Protonephridial), 38 (Neural). (c) Distance comparisons of SpNBs to either corresponding mature tissue of parenchymal cell types. Data from (a) and (b). Two-sided Welch's t-test, * = p=0.0178, *** = p=0.001, **** = p<0.0001. The central line represents the mean and error bars represent standard deviation. (d) Distance measurements of specialized neoblasts in transverse sections after sagittal amputation (5dpa). N = 36 for old tissue, 31 for wound tissue. Note: neural comparisons made to ventral nerve cords. The upper and lower hinges in (a), (b), and (d) correspond to 25th and 75th percentiles, whiskers represent the smallest and largest values, and the line corresponds to the median. Parenchymal and neural data is the same as in Fig. 5g. Source data are provided as a Source Data file.

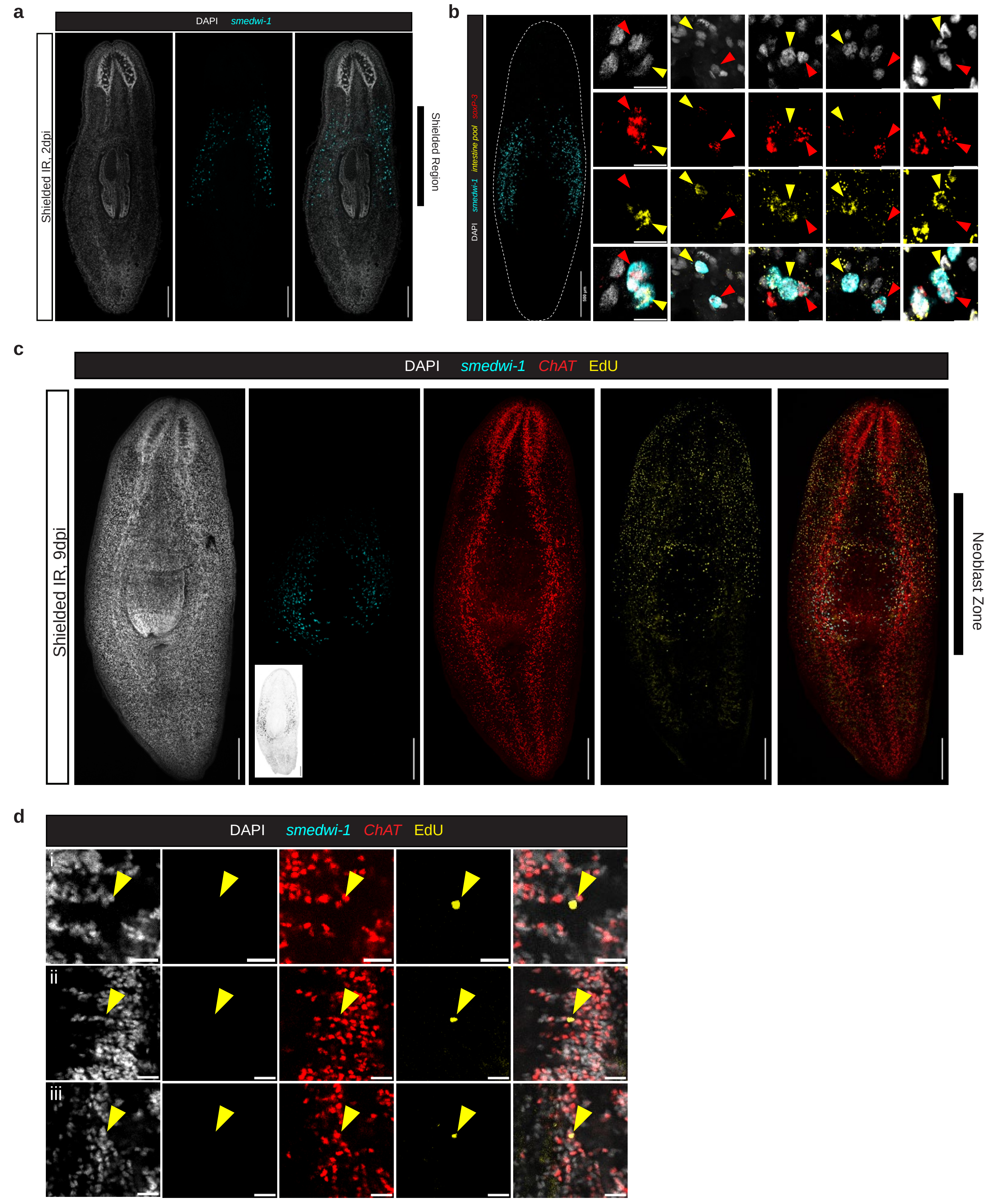
a
DAPI smedwi-1
Shielded IR, 2dpi
Shielded Region
b
DAPI smedwi-1 intestine pool soxP-3
c
DAPI smedwi-1 ChAT EdU
Shielded IR, 9dpi
Neoblast Zone
d
DAPI smedwi-1 ChAT EdU
i
ii
iii

Sup Fig. 31

Supplementary Figure 31. Shielded irradiation of planarians

(a) A lead-shielded animal 2 days post irradiation (dpi). The mid-body of individuals was lead shielded prior to exposure to 30 Gray of unidirectional X-ray exposure. Black bar (right) depicts shielded area. Scale bars, 250 µm. (b) FISH images of intestinal and epidermal specialized neoblasts in shielded regions after irradiation. Scale bars, 500 µm (left), 5 µm (right). First, fourth, and fifth columns (right) contain same images as in Fig. 6b. (c) A lead-shielded animal 9 dpi that was pulsed with EdU 2 dpi. Black bar (right) depicts area in which neoblasts were detected by FISH. Inset depicts inverted and saturated *smedwi-1* fluorescence signal. Images shown are the same as in Fig 6c. Scale bars, 250 µm. (d) Images shown correspond to Fig 6ci-iii. Arrows depict EdU^{+}/$\textit{ChAT}^{+}$ cells. Scale bars, 20 µm.

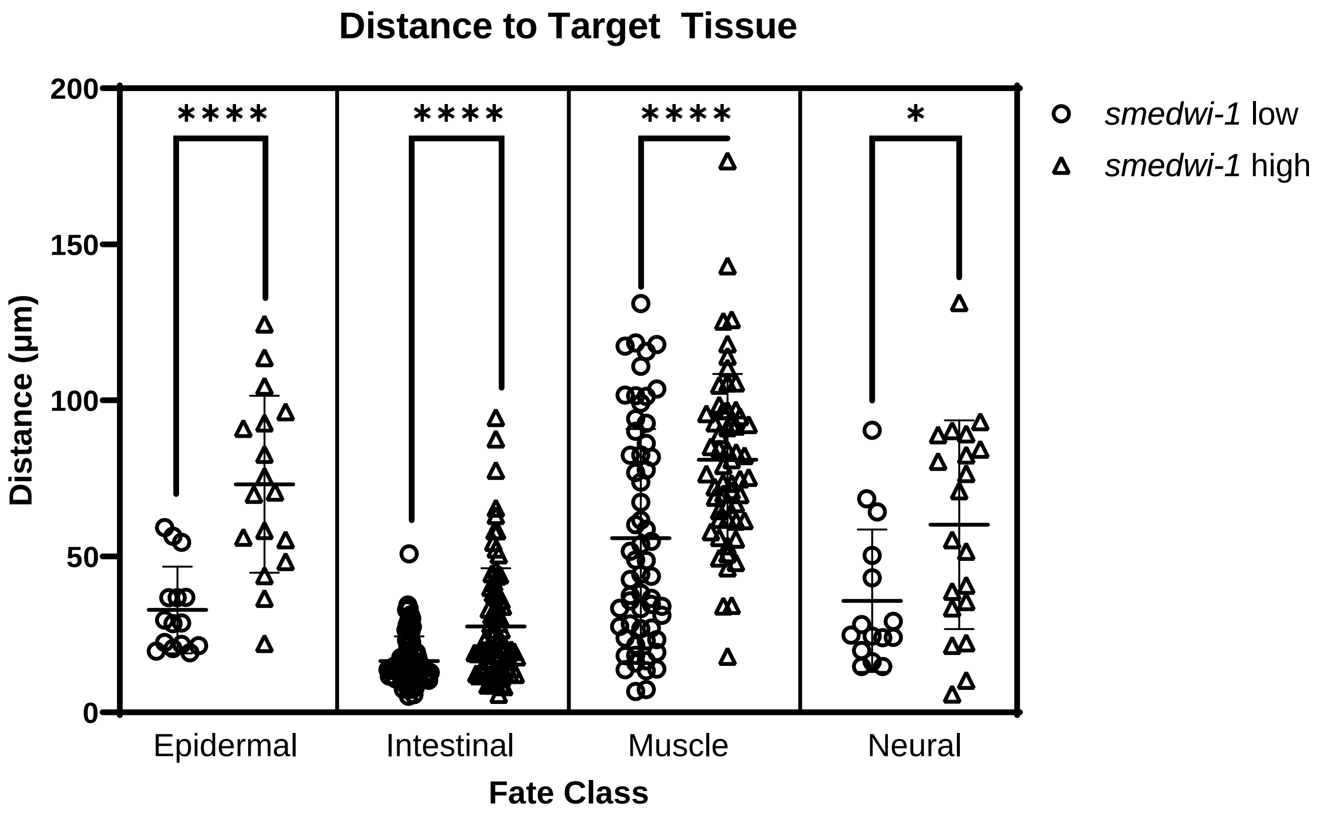

Distance to Target Tissue
200
150
100
50
0
Distance (µm)
****
****
****
*
smedwi-1 low
smedwi-1 high
Epidermal
Intestinal
Muscle
Neural
Fate Class

Sup Fig. 32

Supplementary Figure 32. Distance measurements of *smedwi-1* low and *smedwi-1* high cells to target mature tissues of different fates by MERFISH.
Distance measurements of ventral epidermal, intestinal, BW muscle, and neural specialized neoblasts and their early post-mitotic progenitors by MERFISH. Two-sided Welch's t-test, **** = p<0.0001, * = p=0.0152. N(*smedwi-1* low, *smedwi-1* high) = 15,17 (Epidermal), 77,77 (Intestinal), 60,57 (Muscle), 15,20 (Neural). The central line represents the mean and error bars represent standard deviation. Source data are provided as a Source Data file.

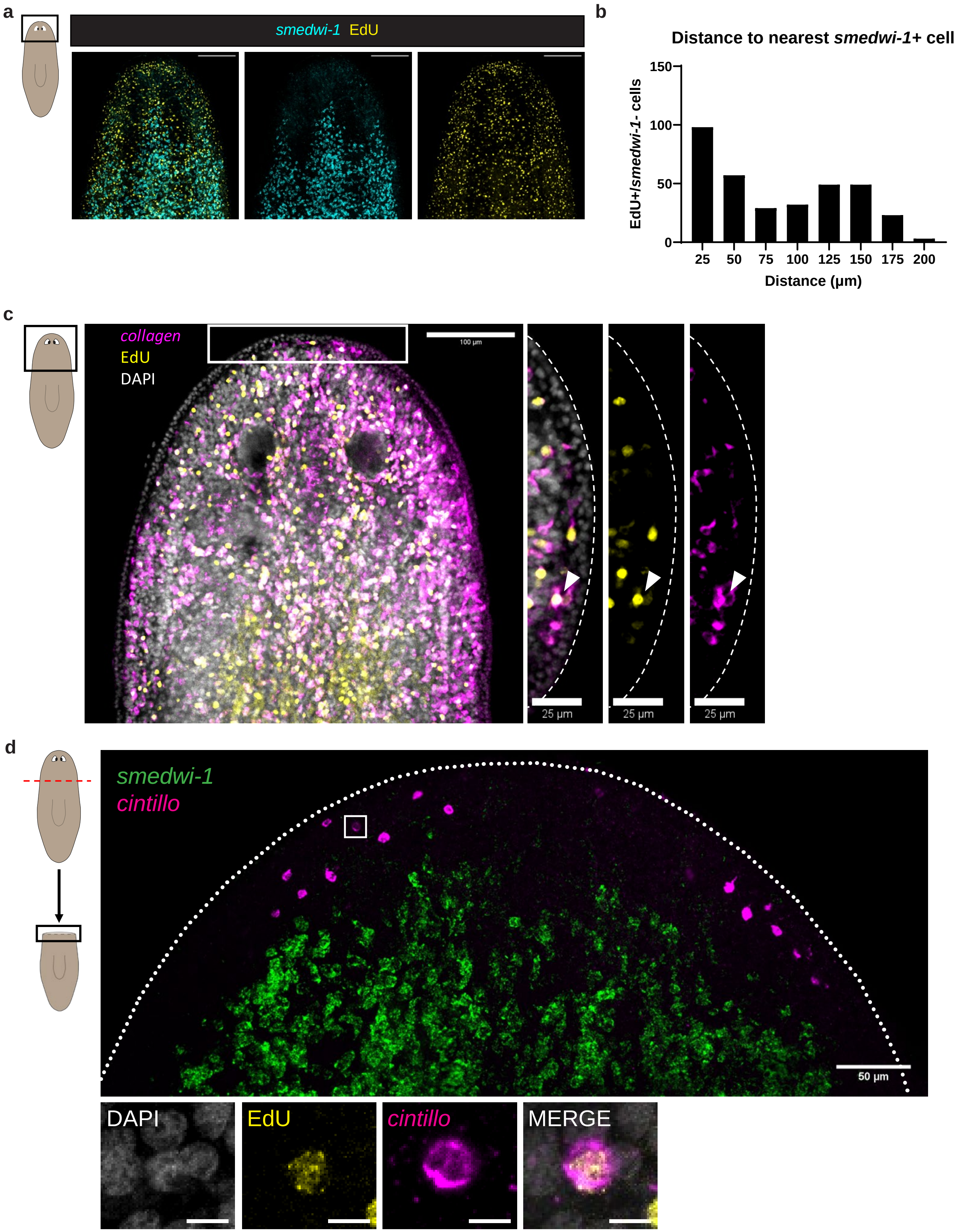

a
smedwi-1 EdU
b
Distance to nearest smedwi-1+ cell
EdU+/smedwi-1- cells
150
100
50
0
25 50 75 100 125 150 175 200
Distance (µm)
c
collagen
EdU
DAPI
100 µm
25 µm
25 µm
25 µm
d
smedwi-1
cintillo
50 µm
DAPI
EdU
cintillo
MERGE

Sup Fig. 33

Supplementary Figure 33. Migratory targeting of progenitors in non-irradiated conditions. (a) EdU+ cells in the anterior head tip. Animals were pulsed with EdU 7 days prior to fixation. Scale bars, 100 µm. (b) Distance measurements of EdU+ cells at the anterior head to past anterior-most neoblast to the closest neoblast. N = 366. (c) EdU incorporation into anterior *collagen*+ muscle cells. Contains same region and images as in Fig. 6e. Scale bars, 100 µm (left), 25 µm (right). (d) EdU incorporation into anterior *cintillo*+ sensory neurons after decapitation. Animals were pulsed with EdU 3dpa and fixed 6dpa. Scale bars, 50 µm (top), 5 µm (bottom). Source data are provided as a Source Data file.

Experiment	Label in this study	Contig
MERFISH	*myoD*	dd_Smed_v6_12634_0_1
MERFISH	*snail*	dd_Smed_v6_10216_0_1
MERFISH	*nkx1.1*	dd_Smed_v6_19327_0_1
MERFISH	*foxD*	dd_Smed_v6_23249_0_1
MERFISH	*zicA*	dd_Smed_v6_22585_0_1
MERFISH	*prep*	dd_Smed_v6_8606_0_1
MERFISH	*gata4/5/6-3*	dd_Smed_v6_8208_0_1
MERFISH	*nk4*	dd_Smed_v6_25344_0_1
MERFISH	*gata4/5/6-2*	dd_Smed_v6_9910_0_1
MERFISH	*foxF-1*	dd_Smed_v6_6910_0_1
MERFISH	*soxP-5*	dd_Smed_v6_9050_0_1
MERFISH	*hnf4*	dd_Smed_v6_1694_0_1
MERFISH	*prox-1*	dd_Smed_v6_13772_0_1
MERFISH	*nkx2.2*	dd_Smed_v6_2716_0_1
MERFISH	*gata4/5/6-1*	dd_Smed_v6_4075_0_1
MERFISH	*RREB2*	dd_Smed_v6_10103_0_1
MERFISH	*lhx2b*	dd_Smed_v6_8190_0_1
MERFISH	*gli-1*	dd_Smed_v6_7470_0_1
MERFISH	*PTF1A*	dd_Smed_v6_6869_0_1
MERFISH	*POU2/3*	dd_Smed_v6_8234_0_1
MERFISH	*six1/2-2*	dd_Smed_v6_9774_0_1
MERFISH	*osr*	dd_Smed_v6_10039_0_1
MERFISH	*sall*	dd_Smed_v6_14176_0_1
MERFISH	*zfp-1*	dd_Smed_v6_8720_0_1
MERFISH	*p53*	dd_Smed_v6_5563_0_1
MERFISH	*soxP-3*	dd_Smed_v6_5942_0_1
MERFISH	*pax6A*	dd_Smed_v6_17726_0_1
MERFISH	*soxB2*	dd_Smed_v6_25321_0_1
MERFISH	*lhx2/9*	dd_Smed_v6_15144_0_1
MERFISH	*foxQ/D*	dd_Smed_v6_50245_0_1
MERFISH	*glass-like*	dd_Smed_v6_75162_0_1
MERFISH	*castor*	dd_Smed_v6_6778_0_1
MERFISH	*elf-1*	dd_Smed_v6_14611_0_1
MERFISH	*lhx3/4*	dd_Smed_v6_16893_0_1
MERFISH	*six3*	dd_Smed_v6_15178_0_1
MERFISH	*soxB*	dd_Smed_v6_13215_0_1
MERFISH	*neuroD-1*	dd_Smed_v6_21717_0_1

MERFISH	*nuclear receptor 1-like (nr-1)*	dd_Smed_v6_94635_0_1
MERFISH	*otxB*	dd_Smed_v6_15516_0_1
MERFISH	*pax6B*	dd_Smed_v6_35892_0_1
MERFISH	*nkx2-like*	dd_Smed_v6_13898_0_1
MERFISH	*nkx6-like*	dd_Smed_v6_11198_0_1
MERFISH	*scratch*	dd_Smed_v6_18952_0_1
MERFISH	*ski-3*	dd_Smed_v6_10394_0_1
MERFISH	*runt-1*	dd_Smed_v6_16222_0_1
MERFISH	*fli-1*	dd_Smed_v6_11113_0_1
MERFISH	*tlx-like*	dd_Smed_v6_13781_0_1
MERFISH	*pax3/7-like (arx)*	dd_Smed_v6_21801_0_1
MERFISH	*ap2*	dd_Smed_v6_15104_0_1
MERFISH	*sim*	dd_Smed_v6_17731_0_1
MERFISH	*hesl-3*	dd_Smed_v6_22479_0_1
MERFISH	*coe*	dd_Smed_v6_9893_0_1
MERFISH	*tcf1*	dd_Smed_v6_13056_0_1
MERFISH	*lhx1/5-1*	dd_Smed_v6_11521_0_1
MERFISH	*pitx*	dd_Smed_v6_15253_0_1
MERFISH	*ovo*	dd_Smed_v6_48430_0_1
MERFISH	*six-1/2-1*	dd_Smed_v6_15436_0_1
MERFISH	*sp6-9*	dd_Smed_v6_17385_0_1
MERFISH	*dlx*	dd_Smed_v6_19040_0_1
MERFISH	*otxA*	dd_Smed_v6_14633_0_1
MERFISH	*eya*	dd_Smed_v6_11372_0_1
MERFISH	*smedwi-1*	dd_Smed_v6_659_0_1
MERFISH	*bruli*	dd_Smed_v6_2592_0_1
MERFISH	*vasa-1*	dd_Smed_v6_1985_0_1
MERFISH	*mcm-7*	dd_Smed_v6_4712_0_1
MERFISH	*soxP-2*	dd_Smed_v6_8483_0_1
MERFISH	*znf333*	dd_Smed_v6_13890_0_1
MERFISH	*H2B*	dd_Smed_v6_2520_0_1
MERFISH	*fgfr-1*	dd_Smed_v6_6020_0_1
MERFISH	*fgfr-4*	dd_Smed_v6_11024_0_1
MERFISH	*soxP-1*	dd_Smed_v6_7227_0_1
MERFISH	*rtel-1*	dd_Smed_v6_12261_0_1
MERFISH	*nanos*	dd_Smed_v6_10484_0_1
MERFISH	*smedwi-2*	dd_Smed_v6_756_0_1
MERFISH	*colF-2*	dd_Smed_v6_702_0_1
MERFISH	*troponin*	dd_Smed_v6_7974_0_1
MERFISH	*tropomyosin*	dd_Smed_v6_436_0_1

MERFISH	dd8528	dd_Smed_v6_8528_0_1
MERFISH	*myosin heavy chain*	dd_Smed_v6_579_0_1
MERFISH	*PRSS12*	dd_Smed_v6_351_0_1
MERFISH	*pbgd*	dd_Smed_v6_626_0_1
MERFISH	*VIT* (dd1071)	dd_Smed_v6_1071_0_1
MERFISH	dd4476	dd_Smed_v6_4476_0_1
MERFISH	*LAMA2* (dd9493)	dd_Smed_v6_9493_0_1
MERFISH	*dd_1320*	dd_Smed_v6_1320_0_1
MERFISH	*mat*	dd_Smed_v6_907_0_1
MERFISH	*cubilin*	dd_Smed_v6_4575_0_1
MERFISH	*ECE2*	dd_Smed_v6_5256_0_1
MERFISH	*CAVII-like*	dd_Smed_v6_4841_0_1
MERFISH	*alkaline phosphatase-1*	dd_Smed_v6_13011_0_1
MERFISH	*Na+/Ca2+ exchanger-like*	dd_Smed_v6_20334_0_1
MERFISH	*HCO3- transporter*	dd_Smed_v6_8250_0_1
MERFISH	*ChAT*	dd_Smed_v6_6208_0_1
MERFISH	*th*	dd_Smed_v6_16581_0_1
MERFISH	*tph*	dd_Smed_v6_8392_0_1
MERFISH	*sert*	dd_Smed_v6_12700_0_1
MERFISH	*pc2*	dd_Smed_v6_1566_0_1
MERFISH	*ITPR3* (dd3814)	dd_Smed_v6_3814_1_1
MERFISH	*trpA*	dd_Smed_v6_14207_0_1
MERFISH	*synapsin*	dd_Smed_v6_3135_0_1
MERFISH	*TTPA* (dd6149)	dd_Smed_v6_6149_0_1
MERFISH	*fer3l-2*	dd_Smed_v6_10868_0_1
MERFISH	*ZAN6* (dd238)	dd_Smed_v6_238_1_1
MERFISH	*glipr-1*	dd_Smed_v6_924_0_1
MERFISH	dd_829	dd_Smed_v6_829_0_1
MERFISH	*X1.A.B7.1*	dd_Smed_v6_750_0_1
MERFISH	*mag-1*	dd_Smed_v6_769_0_1
MERFISH	*FAM115C-like*	dd_Smed_v6_3451_0_1
MERFISH	dd_385	dd_Smed_v6_385_0_1
MERFISH	*SSPO* (dd628)	dd_Smed_v6_628_0_1
FISH	*gata4/5/6-1*	dd_Smed_v6_4075_0_1
FISH	*hnf4*	dd_Smed_v6_1694_0_1
FISH	*pax6A*	dd_Smed_v6_17726_0_1
FISH	*soxP-3*	dd_Smed_v6_5942_0_1
FISH	*ovo*	dd_Smed_v6_48430_0_1
FISH	*POU2/3*	dd_Smed_v6_8234_0_1
FISH	*six-1/2-2*	dd_Smed_v6_9774_0_1
FISH	*foxA*	dd_Smed_v6_10718_0_1

FISH	*myoD*	dd_Smed_v6_12634_0_1
FISH	*snail*	dd_Smed_v6_10216_0_1
FISH	*smedwi-1*	dd_Smed_v6_659_0_1
FISH	*mat*	dd_Smed_v6_907_0_1
FISH	*ChAT*	dd_Smed_v6_6208_0_1

Supplementary Table 1

A list of all genes utilized in this study.

Fate Categorization	Label in this study	Contig
Muscle	*myoD*	dd_Smed_v6_12634_0_1
Muscle	*snail*	dd_Smed_v6_10216_0_1
Muscle	*nkx1.1*	dd_Smed_v6_19327_0_1
Muscle	*foxD*	dd_Smed_v6_23249_0_1
Muscle	*zicA*	dd_Smed_v6_22585_0_1
Muscle	*prep*	dd_Smed_v6_8606_0_1
Muscle	*gata4/5/6-3*	dd_Smed_v6_8208_0_1
Muscle	*nk4*	dd_Smed_v6_25344_0_1
Muscle	*gata4/5/6-2*	dd_Smed_v6_9910_0_1
Muscle	*foxF-1*	dd_Smed_v6_6910_0_1
Muscle	*soxP-5*	dd_Smed_v6_9050_0_1
Intestine	*hnf4*	dd_Smed_v6_1694_0_1
Intestine	*prox-1*	dd_Smed_v6_13772_0_1
Intestine	*nkx2.2*	dd_Smed_v6_2716_0_1
Intestine	*gata4/5/6-1*	dd_Smed_v6_4075_0_1
Intestine	*RREB2*	dd_Smed_v6_10103_0_1
Intestine	*lhx2b*	dd_Smed_v6_8190_0_1
Intestine	*gli-1*	dd_Smed_v6_7470_0_1
Intestine	*PTF1A*	dd_Smed_v6_6869_0_1
Protonephridia	*POU2/3*	dd_Smed_v6_8234_0_1
Protonephridia	*six1/2-2*	dd_Smed_v6_9774_0_1
Protonephridia	*osr*	dd_Smed_v6_10039_0_1
Protonephridia	*sall*	dd_Smed_v6_14176_0_1
Epidermis	*zfp-1*	dd_Smed_v6_8720_0_1
Epidermis	*p53*	dd_Smed_v6_5563_0_1
Epidermis	*soxP-3*	dd_Smed_v6_5942_0_1
Epidermis	*foxJ1-4*	dd_Smed_v6_10152_0_1
Neural	*pax6A*	dd_Smed_v6_17726_0_1
Neural	*soxB2*	dd_Smed_v6_25321_0_1
Neural	*lhx2/9*	dd_Smed_v6_15144_0_1
Neural	*foxQ/D*	dd_Smed_v6_50245_0_1
Neural	*glass-like*	dd_Smed_v6_75162_0_1
Neural	*castor*	dd_Smed_v6_6778_0_1
Neural	*elf-1*	dd_Smed_v6_14611_0_1
Neural	*lhx3/4*	dd_Smed_v6_16893_0_1
Neural	*six3*	dd_Smed_v6_15178_0_1
Neural	*soxB*	dd_Smed_v6_13215_0_1
Neural	*neuroD-1*	dd_Smed_v6_21717_0_1
Neural	*nuclear receptor 1-like (nr-1)*	dd_Smed_v6_94635_0_1
Neural	*otxB*	dd_Smed_v6_15516_0_1

Neural	*pax6B*	dd_Smed_v6_35892_0_1
Neural	*nkx2-like*	dd_Smed_v6_13898_0_1
Neural	*nkx6-like*	dd_Smed_v6_11198_0_1
Neural	*scratch*	dd_Smed_v6_18952_0_1
Neural	*ski-3*	dd_Smed_v6_10394_0_1
Neural	*runt-1*	dd_Smed_v6_16222_0_1
Neural	*fli-1*	dd_Smed_v6_11113_0_1
Neural	*tlx-like*	dd_Smed_v6_13781_0_1
Neural	*pax3/7-like (arx)*	dd_Smed_v6_21801_0_1
Neural	*ap2*	dd_Smed_v6_15104_0_1
Neural	*sim*	dd_Smed_v6_17731_0_1
Neural	*hesl-3*	dd_Smed_v6_22479_0_1
Neural	*coe*	dd_Smed_v6_9893_0_1
Neural	*tcf1*	dd_Smed_v6_13056_0_1
Neural	*lhx1/5-1*	dd_Smed_v6_11521_0_1
Neural	*pitx*	dd_Smed_v6_15253_0_1
Eye	*ovo*	dd_Smed_v6_48430_0_1
Eye	*six-1/2-1*	dd_Smed_v6_15436_0_1
Eye	*sp6-9*	dd_Smed_v6_17385_0_1
Eye	*dlx*	dd_Smed_v6_19040_0_1
Eye	*otxA*	dd_Smed_v6_14633_0_1
Eye	*eya*	dd_Smed_v6_11372_0_1

Supplementary Table 2

FSTF categorization utilized in MERFISH gene pools.

Fate Categorization	Label in this study	Contig
Intestine	*gata4/5/6-1*	dd_Smed_v6_4075_0_1
Intestine	*hnf4*	dd_Smed_v6_1694_0_1
CNS	*pax6A*	dd_Smed_v6_17726_0_1
Epidermis	*soxP-3*	dd_Smed_v6_5942_0_1
Eye	*ovo*	dd_Smed_v6_48430_0_1
Protonephridia	*POU2/3*	dd_Smed_v6_8234_0_1
Protonephridia	*six-1/2-2*	dd_Smed_v6_9774_0_1
Pharynx	*foxA*	dd_Smed_v6_10718_0_1
Muscle	*myoD*	dd_Smed_v6_12634_0_1
Muscle	*snail*	dd_Smed_v6_10216_0_1

Supplementary Table 3

FSTF categorization utilized in whole-mount FISH experiments.

Table S4. Sample Numbers for Figure Datasets

Sample	SpNB Type 1 (cells)	SpNB Type 2 (cells)	Unlabeled (cells)
Eye + CNS Pre-Pharyngeal H	Eye(13)	CNS(476)	Unlabeled(9948)
Eye + Epidermis Pre-Pharyngeal H	Eye(13)	Epidermis(219)	Unlabeled(1615)
Eye + Epidermis AFW R	Eye(38)	Epidermis(698)	Unlabeled(6932)
Eye + Intestine Pre-Pharyngeal H	Eye(22)	Intestine(477)	Unlabeled(3485)
Eye + Intestine AFW R	Eye(23)	Intestine(245)	Unlabeled(856)
Eye + Protonephridia Pre-Pharyngeal H	Eye(13)	Protonephridia(53)	Unlabeled(1927)
CNS + Muscle Pre-Pharyngeal H	*CNS (145)*	Muscle(45)	Unlabeled (417)
CNS + Intestine Pre-Pharyngeal H	*CNS (73)*	Intestine(98)	Unlabeled (469)
CNS + Intestine Post-Pharyngeal H	*CNS (81)*	Intestine(125)	Unlabeled (443)
CNS + Epidermis Pre-Pharyngeal H	*CNS (60)*	Epidermis (195)	Unlabeled (600)
CNS + Epidermis AFW R	CNS(237)	Epidermis(468)	Unlabeled(2084)
CNS + Epidermis Post-Pharyngeal H	*CNS (52)*	Epidermis (134)	Unlabeled (306)
Protonephridia + Intestine Pre-Pharyngeal H	Protonephridia(8)	Intestine(22)	Unlabeled (126)
Protonephridia + Intestine AFW R	Protonephridia(46)	Intestine(176)	Unlabeled(1547)
Protonephridia + Epidermis Pre-Pharyngeal H	Protonephridia(35)	Epidermis (108)	Unlabeled (379)
Protonephridia + Epidermis AFW R	Protonephridia(39)	Epidermis(197)	Unlabeled(1190)
Protonephridia + Epidermis Post-Pharyngeal H	Protonephridia(21)	Epidermis (163)	Unlabeled (398)
Muscle + Protonephridia AFW R	Muscle(40)	Protonephridia(38)	Unlabeled(1157)
Muscle + Intestine Pre-Pharyngeal H	Muscle(34)	Intestine(109)	Unlabeled (590)
Muscle + Intestine AFW R	Muscle(49)	Intestine(145)	Unlabeled(1071)
Muscle + Intestine Post-Pharyngeal H	Muscle(36)	Intestine(87)	Unlabeled (480)
Muscle + Epidermis Pre-Pharyngeal H	Muscle(32)	Epidermis (135)	Unlabeled (469)
Muscle + Epidermis AFW R	Muscle(27)	Epidermis(100)	Unlabeled(824)
Muscle + Epidermis Post-Pharyngeal H	Muscle(12)	Epidermis (169)	Unlabeled (526)
Epidermis + Intestine Post-Pharyngeal H	Epidermis (80)	Intestine(39)	Unlabeled (238)
Epidermis + Intestine Pre-Pharyngeal H	Epidermis (385)	Intestine(255)	Unlabeled (1461)
Epidermis + Intestine AFW R	Epidermis(287)	Intestine(249)	Unlabeled(2142)
Pharynx + CNS Trunk H	Pharynx(215)	CNS(269)	Unlabeled(1169)
Pharynx + Protonephridia Trunk H	Pharynx(119)	Protonephridia(31)	Unlabeled(1009)
Pharynx + Intestine Trunk H	Pharynx(280)	Intestine(658)	Unlabeled(1430)
Pharynx + Epidermis Trunk H	Pharynx(55)	Epidermis(183)	Unlabeled(472)

Key
R = Regeneration
H = Homeostatic
AFW = Anterior Facing Wound
Trunk = Mid-section containing Pharynx

Supplementary Table 4

Specialized neoblast numbers utilized for neighborhood composition analysis.
