# Supplementary material for: Fate specification is spatially intermingled across planarian stem cells
Source: Nat Commun. 2023 Nov 16;14:7422. doi: 10.1038/s41467-023-43267-2 (PMC10654723; doi:10.1038/s41467-023-43267-2)
Supplement: Supplementary file 3 — Description of Additional Supplementary Files [file 41467_2023_43267_MOESM3_ESM.pdf]

Description of Additional Supplementary Files

Supplementary Movie 1: 3D signal visualization of a protonephridial FSTFs (magenta) and smedwi-1 (green) in Imaris

Supplementary Movie 2: Generation of neoblast surfaces (yellow) by smedwi-1 3D signal visualization (cyan) in Imaris

Supplementary Movie 3: Identified protonephridial specialized neoblast surfaces overlayed on 3D signal from protonephridial FSTFs (yellow) and smedwi-1 (cyan)

Supplementary Movie 4: Identified intestinal specialized neoblast surfaces overlayed on 3D signal from intestinal FSTFs (red) and smedwi-1 (cyan)

Supplementary Movie 5: Combined view of Supplementary Movies S2-S4 depicting protonephridial (yellow), intestinal (red), and unidentified neoblast (cyan) surfaces.

Supplementary Movie 6: Intermingled muscle (yellow) and epidermal (red) specialized neoblast surfaces from Figure 3b

Supplementary Movie 7: Intermingled epidermal (red) and protonephridial (yellow) specialized neoblast surfaces from Figure 3b

Supplementary Movie 8: A directly adjacent protonephridial and intestinal specialized neoblast pair from Supplementary Figure 21b. 3D signal visualization of protonephridial FSTFs (yellow), intestinal FSTFs (red), and smedwi-1 (cyan).
