# Supplementary material for: Fate specification is spatially intermingled across planarian stem cells
Source: Nat Commun. 2023 Nov 16;14:7422. doi: 10.1038/s41467-023-43267-2 (PMC10654723; doi:10.1038/s41467-023-43267-2)
Supplement: Supplementary file 12 — Reporting Summary [file 41467_2023_43267_MOESM12_ESM.pdf]

nature portfolio

Corresponding author(s): Dr. Peter Reddien

Last updated by author(s): Oct 24, 2023

Reporting Summary

Nature Portfolio wishes to improve the reproducibility of the work that we publish. This form provides structure for consistency and transparency in reporting. For further information on Nature Portfolio policies, see our Editorial Policies and the Editorial Policy Checklist.

Statistics

For all statistical analyses, confirm that the following items are present in the figure legend, table legend, main text, or Methods section.

n/a	Confirmed	
☐	☒	The exact sample size (*n*) for each experimental group/condition, given as a discrete number and unit of measurement
☐	☒	A statement on whether measurements were taken from distinct samples or whether the same sample was measured repeatedly
☐	☒	The statistical test(s) used AND whether they are one- or two-sided<br>*Only common tests should be described solely by name; describe more complex techniques in the Methods section.*
☒	☐	A description of all covariates tested
☐	☒	A description of any assumptions or corrections, such as tests of normality and adjustment for multiple comparisons
☐	☒	A full description of the statistical parameters including central tendency (e.g. means) or other basic estimates (e.g. regression coefficient) AND variation (e.g. standard deviation) or associated estimates of uncertainty (e.g. confidence intervals)
☐	☒	For null hypothesis testing, the test statistic (e.g. *F*, *t*, *r*) with confidence intervals, effect sizes, degrees of freedom and *P* value noted<br>*Give P values as exact values whenever suitable.*
☒	☐	For Bayesian analysis, information on the choice of priors and Markov chain Monte Carlo settings
☒	☐	For hierarchical and complex designs, identification of the appropriate level for tests and full reporting of outcomes
☒	☐	Estimates of effect sizes (e.g. Cohen's *d*, Pearson's *r*), indicating how they were calculated

Our web collection on statistics for biologists contains articles on many of the points above.

Software and code

Policy information about availability of computer code

Data collection	Data for MERFISH experiments were collected using proprietary MERSCOPE software (Vizgen).
Data analysis	Python libraries applied in Voronoi analysis include: freud v2.13.l, Matplotlib v3.8.0, NumPy vl.26, plato v0.2.1, pandas v2.l.0, as well as the Python v3.ll.5 Standard Library. Nearest neighbor analysis and simulations were performed in MATLAB R2023b.<br>Image analysis: ImageJ v1.54, MERSCOPE Visualizer v2.2.3002.0, Imaris v10.0.0, QuPATH v0.4.2

For manuscripts utilizing custom algorithms or software that are central to the research but not yet described in published literature, software must be made available to editors and reviewers. We strongly encourage code deposition in a community repository (e.g. GitHub). See the Nature Portfolio guidelines for submitting code & software for further information.

Data

Policy information about availability of data

All manuscripts must include a data availability statement. This statement should provide the following information, where applicable:

- Accession codes, unique identifiers, or web links for publicly available datasets
- A description of any restrictions on data availability
- For clinical datasets or third party data, please ensure that the statement adheres to our policy

All data supporting the findings of this study are available in the article or Supplementary Information. Source data are provided with this paper.

Research involving human participants, their data, or biological material

Policy information about studies with human participants or human data. See also policy information about sex, gender (identity/presentation), and sexual orientation and race, ethnicity and racism.

Reporting on sex and gender	Not applicable for this study.
Reporting on race, ethnicity, or other socially relevant groupings	Not applicable for this study.
Population characteristics	Not applicable for this study.
Recruitment	Not applicable for this study.
Ethics oversight	Not applicable for this study.

Note that full information on the approval of the study protocol must also be provided in the manuscript.

Field-specific reporting

Please select the one below that is the best fit for your research. If you are not sure, read the appropriate sections before making your selection.

☒ Life sciences ☐ Behavioural & social sciences ☐ Ecological, evolutionary & environmental sciences

For a reference copy of the document with all sections, see nature.com/documents/nr-reporting-summary-flat.pdf

Life sciences study design

All studies must disclose on these points even when the disclosure is negative.

Sample size	For FISH and MERFISH experiments, sample size was not determined prior to the experiment.
Data exclusions	No data were excluded from this study.
Replication	All experimental findings were reproduced in at least 2 independent experiments.
Randomization	All animals utilized for experiments were selected randomly from a large collection of clonal animals.
Blinding	Investigators were not blinded during data collection and analysis as all different conditions tested were visible to the naked eye (regenerating blastemas, uninjured animals, sublethally irradiated animals).

Reporting for specific materials, systems and methods

We require information from authors about some types of materials, experimental systems and methods used in many studies. Here, indicate whether each material, system or method listed is relevant to your study. If you are not sure if a list item applies to your research, read the appropriate section before selecting a response.

Materials & experimental systems

n/a	Involved in the study	
☐	☒	Antibodies
☒	☐	Eukaryotic cell lines
☒	☐	Palaeontology and archaeology
☐	☒	Animals and other organisms
☒	☐	Clinical data
☒	☐	Dual use research of concern
☒	☐	Plants

Methods

n/a	Involved in the study	
☒	☐	ChIP-seq
☒	☐	Flow cytometry
☒	☐	MRI-based neuroimaging

Antibodies

Antibodies used	Commercial antibodies were utilized in signal development for fluorescence in situ hybridization experiments. Anti-Digoxigenin-POD, Fab fragments (1:1000, Roche #11207733910), Anti-Fluorescein-POD, Fab fragments (1:2000, Roche #11426346910), Anti-DNP HRP (1:100, Perkin Elmer #FP1129).

Validation	Listed antibodies are utilized commonly in planarian studies. Validation:<br>Cote, L. et al. Muscle functions as a connective tissue and source of extracellular matrix in planarians. Nature Communications 10, 1592 (2019).

Animals and other research organisms

Policy information about studies involving animals; ARRIVE guidelines recommended for reporting animal research, and Sex and Gender in Research

Laboratory animals	Asexual Schmidtea mediterranea (Clone CW4). Asexual planaria are clonal and relies on fission for reproduction. Asexual planaria are not known to age.
Wild animals	This study did not include wild caught animals.
Reporting on sex	Animals utilized in this study are asexual and clonally derived.
Field-collected samples	This study did not utilize any samples collected in the field.
Ethics oversight	No ethics approval is required as planarians are unregulated marine invertebrate animals.

Note that full information on the approval of the study protocol must also be provided in the manuscript.
